# Evaluating Large Language Models in Clinical Audiology (AUDIOLOGYBENCH): Benchmark Development and Validation Study

**DOI:** 10.2196/94755

**Published:** 2026-09-11

**Authors:** Linkai Li, Changgeng Mo, Haoshuai Zhou, Hanlin Yu, Congxi Lu, Shangqiguo Wang, Varsha M Athreya, Matthew B Fitzgerald, Shan X Wang

**Affiliations:** 1 Department of Electrical Engineering, Stanford University, Stanford, CA, United States; 2 Orka Labs Inc, Shanghai, China; 3 Department of Electrical and Computer Engineering, University of British Columbia, Vancouver, BC, Canada; 4 Faculty of Education, University of Hong Kong, Hong Kong, Hong Kong, China (Hong Kong); 5 Department of Otolaryngology-Head and Neck Surgery, Stanford University, Stanford, CA, United States; 6 Department of Electrical Engineering, Stanford University, Address: McCullough Building, Room 351, 476 Lomita Mall, Stanford, CA, United States, 1 650-723-8671

**Keywords:** large language models, audiology, clinical decision support, benchmarking, AI

## Abstract

**Background:**

Large language models (LLMs) are increasingly being explored for clinical decision support, but their performance in audiology has not been systematically benchmarked using clinically grounded case materials and rubric-based safety evaluations.

**Objective:**

This study aimed to develop and evaluate AUDIOLOGYBENCH, a 3-tier benchmark for characterizing frontier LLM capability in clinical audiology along (1) curated domain knowledge, (2) literature-derived evidence, and (3) clinical reasoning under multimodal case input, with an explicit human audit of the automated adjudicator on the primary end point.

**Methods:**

The benchmark comprises 3139 objective items from educational resources, 3175 research article–derived items from peer-reviewed articles published between 2015 and 2025, and 67 multimodal clinical case studies graded against a standardized A-F rubric with 6 prespecified critical-error types that cap scores at D or F. Eight models were evaluated on the educational objective items: 4 frontier multimodal models (Gemini 2.5 Pro, Grok 4, OpenAI O3, and Claude Sonnet 4 Thinking) were evaluated on the research article–derived items, and on 804 case study evaluations. Adjudication used Gemini 2.5 Pro (objective and research-derived items) and Claude Opus 4.5 (case studies). The case study adjudicator was independently audited against PhD-level audiologist consensus on blinded subsamples, supplemented by a post-stratified human-calibrated sensitivity analysis.

**Results:**

A striking task-type dissociation emerged on case studies: clinical recommendations (Q3) achieved a mean score of 89.74 (SD 13.92, 95% CI 88.07‐91.41), a 98.1% (263/268) pass rate, and no dangerous recommendations; audiometric numerical interpretation (Q1) achieved a mean score of 67.89 (SD 18.47, 95% CI 65.68‐70.10), with a 35.4% (95/268) critical-error rate; and differential diagnosis (Q2) achieved a mean score of 67.79 (SD 15.33, 95% CI 65.95‐69.63). Question type, not model selection, dominated performance (eta-squared_H=0.333 vs 0.001; rank biserial *r*≥0.679). Interreviewer reliability between audiologists was high (quadratic-weighted κ of 0.78 and 0.85 across the 80-item and 50-item audits, respectively). When 2 audiologists regraded all 80 model Q1 responses with the diagnostic images available, the adjudicator’s per-item Q1 labels diverged from human judgment (κ=0.05; overflagging; sensitivity: 19/26, 73%; positive predictive value: 19/53, 36%), yet its reweighted Q1 critical-error rate (36.2%) was broadly consistent with the image-grounded human estimates (28%‐34%), suggesting no systematic inflation of the headline rate. The principal Q3>{Q1, Q2} ranking was preserved under post-stratified human calibration. Web-style multiple-choice items showed ceiling effects (>95% accuracy); short-answer prompts remained challenging (best 30%).

**Conclusions:**

Current frontier LLMs show strong recommendation generation but substantial limitations in audiometric numerical interpretation that are shared across models and that an automated adjudicator partially miscalibrated at the per-item level. AUDIOLOGYBENCH characterizes capability boundaries rather than certifying clinical readiness. Deployment of LLM-assisted audiology workflows requires structured human verification of all numerical findings and awareness of fabrication and severity misclassification failure modes documented here.

## Introduction

Large language models (LLMs) have become increasingly prominent in health care delivery and clinical decision support. Since ChatGPT (OpenAI) first approached the passing threshold on the United States Medical Licensing Examination (USMLE) questions in late 2022, progress has been rapid. Contemporary frontier models now achieve expert-level performance on medical licensing examinations, with Med-Gemini (Google) reaching 91.1% accuracy on the MedQA benchmark [1] and GPT-4 exceeding the USMLE passing threshold by over 20 points [2]. This rapid advancement has catalyzed widespread adoption: according to the American Medical Association surveys, physician AI usage has nearly doubled from 38% in 2023 to 66% in 2024 [3].

Yet deployment in specialized clinical settings demands more than strong performance on broad medical question answering (QA): it requires domain-specific validity and up-to-date knowledge [2,4,5]. Audiology (encompassing diagnosis, device fitting, counseling, and longitudinal care) exemplifies this need [6]. The discipline demands precise numerical interpretation of audiometric data, including pure-tone thresholds across multiple frequencies, speech recognition thresholds, and word recognition scores. Errors of even 5 to 10 dB can alter clinical classification and change management decisions. Diagnostic reasoning in audiology requires systematic integration of these quantitative findings with patient history, tympanometric results, and acoustic reflex patterns to differentiate among conductive, sensorineural, and mixed hearing losses across a spectrum of severity classifications.

The medical LLM evaluation landscape has expanded rapidly, moving from examination-style benchmarks, such as MedQA [4] and MedMCQA [7], to comprehensive clinical evaluation frameworks, including HealthBench [8] and MedHELM [9], with dedicated evaluations emerging across specialties, including ophthalmology [10] and non-English clinical settings [11]. Yet audiology remains largely underexplored in this landscape. The most directly relevant prior work comes from Wang et al [12], who evaluated GPT-4 on 299 multiple-choice questions from the Taiwan audiologist qualification examination, reporting 75% overall accuracy, with performance varying from 88% on basic auditory science to only 58% on electrophysiological audiology. More recently, Karaçaylı et al [13] evaluated multimodal LLMs on pure-tone audiogram interpretation, comparing ChatGPT-5.0 and Gemini 2.5 on 80 audiograms for diagnostic accuracy. However, both studies used structured formats (multiple-choice questions or single-task audiogram interpretation) that do not capture the complexity of comprehensive clinical reasoning. No published benchmark has systematically evaluated LLM performance on open-ended clinical audiology case analysis with safety-graded outputs across multiple frontier models.

We present AUDIOLOGYBENCH, a multitier benchmark that evaluates LLMs along three dimensions: (1) curated domain knowledge through objective items (multiple-choice questions [MCQ], true or false [TF], and fill-in-the-blank [FIB]) from educational resources; (2) literature-derived evidence via items adapted from peer-reviewed articles published between 2015 and 2025, testing familiarity with published research findings (including short-answer [SA] prompts requiring precise factual extraction); and (3) clinical reasoning through case studies evaluated with a standardized grading framework incorporating critical-error classification. Our results demonstrate that near-ceiling accuracy on web-style MCQs can mask substantial weaknesses, SA prompts remain difficult, and clinical case analysis reveals a striking dissociation between strong recommendation generation and weak audiometric interpretation.

The benchmark was designed to test 3 prespecified hypotheses:

- Hypothesis 1. Web-style multiple-choice items will exhibit ceiling effects across frontier LLMs.
- Hypothesis 2. Short-answer and rubric-graded case formats will be substantially more discriminative than format-saturated MCQ or TF items.
- Hypothesis 3: Performance differences across task types will exceed performance differences across models, indicating shared limitations of current frontier LLMs rather than model-specific artifacts.

## Methods

### Ethical Considerations

All materials were drawn from publicly available audiology resources (examinations, educational resources, case repositories, and peer-reviewed articles). Cases were anonymized and standardized; no patient records or interactions were used. The study involved only deidentified, public data and did not constitute human subjects research; therefore, institutional review board review was not required. The expanded Q1 audit and the within-task human baseline were performed by coauthor audiologists (study team), blinded to model identity, consistent with this determination.

### Evaluation Framework

The benchmark evaluates LLMs along 3 tiers with a defined end point hierarchy. The primary end point is the clinical case rubric grade (A to F) and critical-error rate, which directly assess clinical interpretability and safety. The secondary end points include (1) objective QA accuracy on educational items, providing baseline domain knowledge coverage; (2) literature-derived QA accuracy, testing evidence familiarity; and (3) automated-adjudicator score components across question types. Objective and literature-derived items serve primarily to validate that the benchmark provides discriminative capacity beyond ceiling-dominated formats. The clinical case rubric constitutes the primary evaluation because it most closely approximates the demands of real clinical workflows in audiology.

### Item Generation and Curation

#### Overview

**Figure 1.**
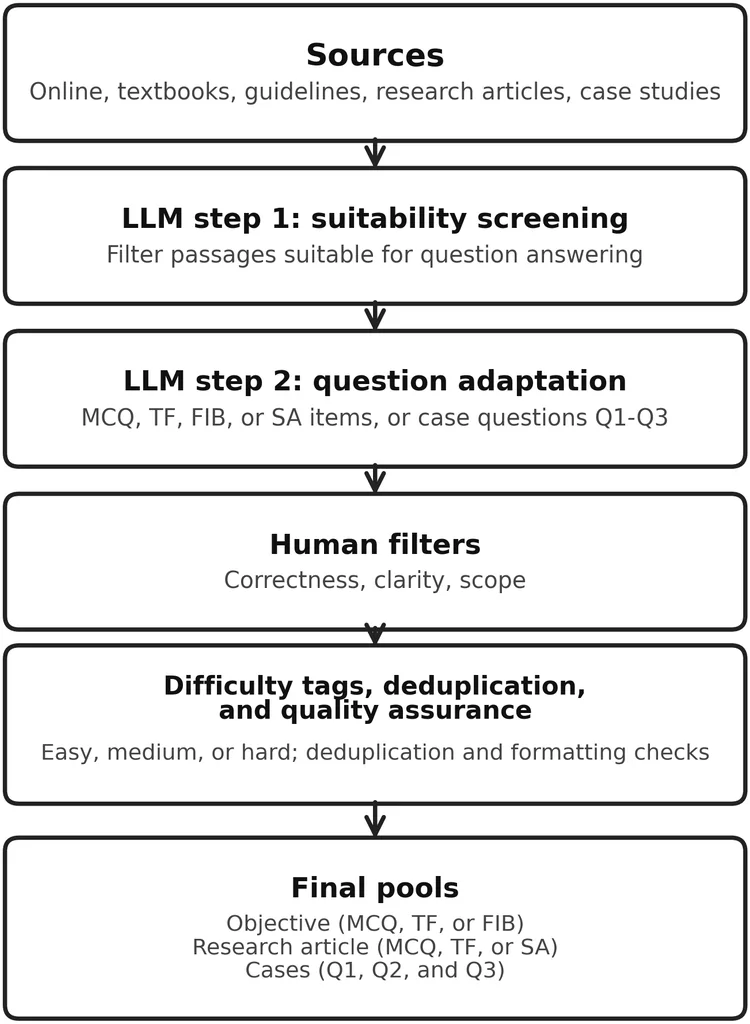
Question generation and curation pipeline. Source materials underwent 2-stage large language model (LLM)–based suitability screening and item adaptation. All pools underwent human review, deduplication, and formatting normalization; objective and research article–derived items also received difficulty tags. The pipeline yielded 3 evaluation pools with distinct competence targets. FIB: fill-in-the-blank; MCQ: multiple-choice question; Q1: interpretation of audiometric results; Q2: diagnostic impression; Q3: clinical recommendations; SA: short answer; TF: true or false.

An overview of dataset construction is shown in Figure 1. The benchmark comprises 3 distinct evaluation pools with different competence targets.

Items at all 3 tiers were processed through the uniform backend pipeline shown in Figure 1. After LLM-based screening and adaptation, every candidate item passed through (1) deduplication by exact match and shingled-overlap detection on item stems and option sets, (2) formatting normalization (option-letter casing, single-best-answer enforcement for MCQ, and unit- and decimal-place standardization on FIB), and (3) difficulty tagging based on cognitive-level and pilot-run accuracy; the difficulty tag is released as metadata for the objective and research-derived pools. Representative items for each format×source pool combination are provided in section N.12 in Multimedia Appendix 1.

#### Objective Items (Educational Resources)

**Figure 2.**
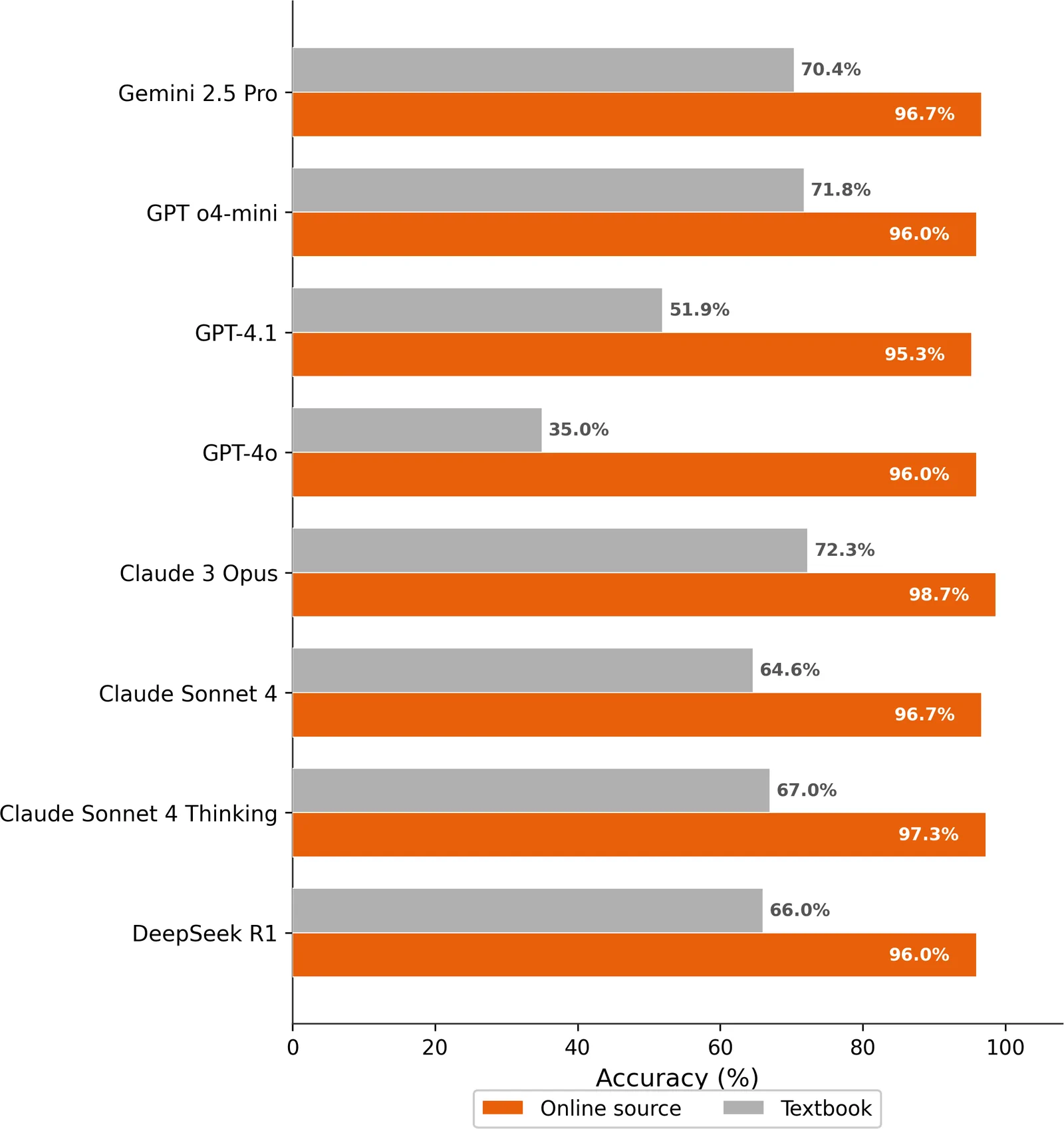
Online versus textbook multiple-choice question (MCQ) accuracy by model. Web-style MCQ exhibits ceiling effects; curated sources remain discriminative.

We curated 3139 items from a combination of three sources: (1) publicly available licensure-preparation materials, (2) open educational websites and QA repositories tagged for audiology and hearing science, and (3) chapter-end question sets and review questions from established audiology textbooks and casebooks. Source titles in (3) were all published from 2015 onward and were selected for coverage of core audiology curricular domains—audiometric interpretation, hearing loss type and severity classification, differential diagnosis, and rehabilitation principles—rather than for recency of publication. The curated set comprised 356 MCQs, 1112 TF items, and 1671 FIB items. The licensure-preparation and open-educational-website pools constitute the “online” source pool referenced in *Objective Items: Online vs Educational Resources* section (Figure 2); the textbook and casebook pool constitutes the “nonweb” source pool. Using Gemini 2.5 Pro (temperature 0), passages were screened and adapted into candidate items. Heuristic filters removed duplicates and near-duplicates, enforced MCQ option balance, and flagged ambiguous stems. Two audiology experts (the corresponding author and one additional doctoral-level audiology reviewer) reviewed candidate items for accuracy, clarity, and scope; flagged items were revised or removed before release.

The percentage distribution across 6 clinically relevant domains was as follows: anatomy and physiology, 29.1%; audiology professional knowledge, 50.7%; clinical decision-making, 11.2%; empathy and communication, 1.7%; instrumentation, 5.9%; and others, 1.4%.

#### Research Article–Derived Items

To evaluate familiarity with published research findings, we converted 3175 items from peer-reviewed audiology, speech, and hearing sciences research articles published between 2015 and 2025: 529 (16.7%) MCQs, 1186 (37.4%) TF, and 1460 (46%) SA questions. A 2-step prompt pipeline with Gemini 2.5 Pro (temperature 0) was used: (1) suitability screening—determine whether a passage can be faithfully transformed into a question with an unambiguous answer; and (2) question adaptation—generate an MCQ, TF, or SA item with a reference answer and minimal rationale. Two audiology experts reviewed the items and reference answers; flagged items were revised or removed. Limitations of this concurrent (vs blinded second pass) review are discussed in section N.13.1 in Multimedia Appendix 1.

Binary item formats are included to provide domain coverage across fundamental audiology concepts. However, binary formats are known to saturate for frontier LLMs owing to the constrained response space [2]. We therefore rely on SA and clinical case tasks as the primary discriminative instruments of this benchmark.

#### Clinical Case Studies

We assembled 67 clinically grounded case studies from established educational resources. Cases represented commonly encountered clinical presentations in audiology practice, including various types and degrees of hearing loss, asymmetric presentations, and cases requiring urgent referral. Each case comprised a text-based case history together with diagnostic images (audiograms, tympanograms, otoacoustic emission results, auditory brainstem response waveforms, and other clinical test outputs); the textual case history was supplied to the evaluated models as text (standard tokenized input), and only the diagnostic images were supplied as image inputs, matching the input composition described in the *Image Handling and Multimodal Adjudication* section and the evaluated model system prompt in section N.1.5 in Multimedia Appendix 1.

Dataset characteristics are summarized in Table 1.

**Table 1.** Case study dataset characteristics^a^.

Parameter	Value
Total cases	67
Data sources	Established audiology textbooks
Models evaluated	Gemini 2.5 Pro, Grok 4, OpenAI O3, and Claude Sonnet 4 Thinking
Question types	3 (Q1: results, Q2: diagnosis, and Q3: recommendations)
Total evaluations	804 (67 cases×4 models×3 questions)

a Each case was paired with 3 standardized prompts: Q1 (audiometric results) for interpretation and reporting of audiometric findings; Q2 (diagnostic impression) for formulation of clinical diagnosis; and Q3 (clinical recommendations) for generation of appropriate management recommendations.

### Model Selection and End Point Hierarchy

The benchmark uses 2 model panels matched to the 2 end point tiers. The educational objective item subtier (3139 items) was evaluated across 8 models (Claude 3 Opus, Claude Sonnet 4, Claude Sonnet 4 Thinking, GPT o4-mini, GPT-4.1, GPT-4o, Gemini 2.5 Pro, and DeepSeek R1) chosen to span the major frontier model families that were commercially available with stable API access during the August 2025 evaluation window. The research article–derived subtier (3175 items) and the case study tier (804 multimodal evaluations) were evaluated across 4 frontier multimodal models (Gemini 2.5 Pro, Grok 4, OpenAI O3, and Claude Sonnet 4 Thinking) chosen because each (1) supported direct image input under its public API, (2) was a top-tier frontier model in the corresponding family during the evaluation window, and (3) provided sufficiently stable API access for the evaluation cost. Models without public multimodal image input support during the evaluation window were not eligible for the case study tier; this excluded DeepSeek R1 (text only at the time of evaluation) and several smaller open-source models. Cost feasibility additionally excluded otherwise eligible models that would have required prohibitive expenditure under the 804-evaluation budget.

The end point hierarchy is as follows: the primary end point was the rubric-graded clinical case scores (804 evaluations across 4 models), capturing clinical reasoning under multimodal input; and the secondary end points were (1) objective item accuracy on educational resources (8 models) and (2) research article–derived item accuracy (4 models). Inferential statistical testing (Kruskal-Wallis, Mann-Whitney *U* with Bonferroni correction, and mixed effects sensitivity analyses) is reserved for the primary end point, where the design supports formal cross-model and cross-task contrasts. The secondary end points are reported descriptively to characterize domain coverage capability and to validate that the benchmark provides discriminative capacity beyond ceiling-dominated formats.

### Models and Inference Protocol

Evaluated model API access for both panels was performed during August 2025 under each provider’s default API settings; exact API snapshot identifiers are reported in Table 2. The case study adjudicator (Claude Opus 4.5; claude-opus-4-5-20251101) was applied to the frozen August 2025 case study responses following its public release in November 2025. The 2 automated adjudicators (Gemini 2.5 Pro for objective and research article–derived items and Claude Opus 4.5 for the 804 clinical case study evaluations) were both run at temperature 0. Single-pass design rationale is provided in the *Limitations* section. On the case study tier, the 4 evaluated models received an identical system prompt establishing a board-certified audiologist persona and requesting a 3-section response (results, diagnosis, and recommendations; ≤150 words each); the verbatim prompt is in section N.1.5 in Multimedia Appendix 1.

**Table 2.** Models and API snapshot identifiers.

Model	Vendor	API snapshot identifier	Tier
Gemini 2.5 Pro	Google	gemini-2.5-pro^a^	Educational^b^+research derived^c^+case study^d^
Grok 4	xAI	grok-4‐0709^e^	Research derived+case study
OpenAI O3	OpenAI	o3-2025-04-16^e^	Research derived+case study
Claude Sonnet 4 Thinking	Anthropic	claude-sonnet-4‐20250514 (extended thinking)^e^	Educational+research derived+case study
Claude Sonnet 4	Anthropic	claude-sonnet-4‐20250514^e^	Educational
Claude 3 Opus	Anthropic	claude-3-opus-20240229^e^	Educational
GPT o4-mini	OpenAI	o4-mini-2025-04-16^e^	Educational
GPT-4.1	OpenAI	gpt-4.1-2025-04-14^e^	Educational
GPT-4o	OpenAI	gpt-4o-2024-11-20^e^	Educational
DeepSeek R1	DeepSeek	deepseek-reasoner^a^	Educational
Claude Opus 4.5	Anthropic	claude-opus-4-5-20251101^e^	Adjudicator^f^ (case study)

a Vendor-provided stable aliases.

b Eight-model educational objective tier.

c Four-model research article–derived tier.

d Four-model multimodal case study tier.

e Date-pinned snapshots.

f Automated adjudicator for the primary end point.

### Response Capture and Answer Extraction

We obtained model responses interactively through each provider’s chat or API end point rather than through a fully automated batched pipeline. For each item, we submitted the standardized prompt and recorded the complete response verbatim. For objective items (MCQ, TF, or FIB), the final selected answer was extracted from the model response following a predefined protocol: for MCQ, the model was prompted to indicate a single option letter, and the first letter matching a valid option in the response was recorded as the selected answer; for TF, the first occurrence of “true” or “false” (case insensitive) was recorded; and for FIB, the answer span identified by the model as filling the blank was recorded verbatim and adjudicated by Gemini 2.5 Pro at temperature 0 against the reference answer using a deterministic binary rubric permitting spelling variants, synonyms, and standard unit forms. When a model response was ambiguous or contained more than one candidate answer for an objective item, the item was resubmitted with a clarifying instruction to provide a single answer, and the resubmitted response was used. MCQ and TF items were scored by deterministic exact match against the reference; the automated-adjudicator path (Gemini 2.5 Pro at temperature 0) was applied to FIB items. For research article–derived SA items, the full response text was passed to the Gemini 2.5 Pro adjudicator without preextraction and adjudication proceeded against the reference answer under the same deterministic binary rubric. For clinical case studies, the complete model response was passed to the Claude Opus 4.5 adjudicator without preextraction. Extraction protocols and adjudication prompt templates are documented in sections N.1.4 to N.1.7 in Multimedia Appendix 1.

### Scoring and Preprocessing

#### Objective Items

MCQ and TF were scored by exact match to the reference answer. FIB and SA responses were adjudicated by Gemini 2.5 Pro at temperature 0 using a deterministic binary rubric permitting spelling variants, synonyms, and standard unit forms. A subset of responses was reviewed by a certified audiologist to check for systematic scoring errors; no major discrepancies were identified in the reviewed samples. Formal interrater reliability metrics (eg, Cohen κ) between human and automated judgments for these objective and research-derived items were not computed; this point is addressed in section N.13.8 in Multimedia Appendix 1. (Interreviewer and adjudicator vs consensus reliability for the case study primary end point is reported separately in the *Human Audit of Automated Adjudication* section.)

#### Clinical Case Studies (Grading Rubric)

For clinical case studies, we developed a comprehensive evaluation framework with domain-specific criteria. Q1 (audiometric results) criteria included numerical accuracy (40%), test coverage (30%), description accuracy (20%), and absence of fabricated audiometric findings (10%). Q2 (diagnostic impression) criteria included diagnostic accuracy (50%), clinical reasoning quality (30%), and evidence-based support (20%). Q3 (clinical recommendations) criteria included relevance to diagnosis (40%), clinical feasibility (30%), and completeness (30%). Rubric weights were determined by expert consensus, reflecting the relative clinical importance of each evaluation dimension.

The asymmetric cap (missed red flag→D; dangerous recommendation→F) preserves the clinical distinction between omission (failure to escalate, recoverable by the next clinician in the workflow) and commission (an active recommendation that, if followed, produces patient harm). Both categories are clinically serious.

All case study responses were evaluated programmatically using Claude Opus 4.5 (temperature 0) as the automated adjudicator. For each evaluation, the adjudicator received the textual reference answer, the model’s response, and the complete scoring rubric (Tables 3, 4, 5) and was instructed to evaluate each rubric subdimension on a 1‐ to 5-point scale and to flag any critical errors from the 6 prespecified categories. Diagnostic images (audiograms, tympanograms, otoacoustic emission results, auditory brainstem response waveforms, and other clinical test outputs) were provided as direct image inputs to the 4 evaluated models but were not provided to the adjudicator; adjudication was based on textual comparison between the model response and the reference answer. The implications of this reference answer–based adjudication design for fabrication detection are discussed in the *Image Handling and Multimodal Adjudication* section and in the *Limitations* section. The approach follows established practices in LLM benchmarking, where automated evaluation enables consistent application of standardized criteria across large-scale assessments [14]. For each evaluation, Claude Opus 4.5 returned a structured JSON object containing per-subdimension scores on a 1‐ to 5-point scale (Q1: numerical accuracy, test coverage, description accuracy, and hallucination-free; Q2: diagnostic accuracy, reasoning logic, and evidence support; and Q3: relevance, clinical feasibility, and completeness), the list of any critical errors of the 6 prespecified types, a brief justification, and an overall assessment label. The 0 to 100 weighted score, A to F letter grade, and score capping rule (for items containing a critical error of a category capped at D or F per Table 4) were then computed deterministically from this JSON output using the rubric weights in Table 5 and the post-adjudication algorithm documented in section N.1.8 in Multimedia Appendix 1. All output components for the full 804-evaluation set are publicly available at the project repository [15]. The verbatim case study adjudicator prompt template (system message, user message template, per-question-type substituted criteria, dimension-score JSON templates, and the post-adjudication deterministic score computation algorithm) is documented in section N.1.8 in Multimedia Appendix 1.

Reference answers were extracted by Gemini 2.5 Pro at temperature 0 under a no-hallucination constraint and reviewed by a doctoral-level audiologist (the corresponding author) for clinical accuracy and source-grounding before the reference set was frozen for adjudication; the verbatim extraction prompt and reviewer attestation note are in section N.1.4 in Multimedia Appendix 1.

**Table 3.** Grading rubric.

Grade	Score range	Description
A	90‐100	Fully correct—exact match or clinically equivalent response
B	75‐89	Mostly correct—minor noncritical errors only
C	50‐74	Partially correct—mix of correct and incorrect elements
D	25‐49	Mostly incorrect—major errors or significant omissions
F	0‐24	Fully wrong—contradicts reference standard or clinically harmful

**Table 4.** Critical-error types and score caps.

Error type	Description	Max grade
Type misclassification	Incorrect hearing loss type (eg, SNHL^a^ as CHL^b^)	D
Severity misclassification	Degree differs by ≥2 categories	D
Fabricated or erroneous test result	Audiometric values or test outcomes not present in or contradicting case materials	D
Laterality confusion	Left and right ear data swapped or misattributed	D
Missed red flag	Failure to identify urgent conditions	D
Dangerous recommendation	Clinically harmful or contraindicated advice	F

a SNHL: sensorineural hearing loss.

b CHL: conductive hearing loss.

**Table 5.** Rubric subdimension definitions^a^.

Q-type	Subdimension	Weight (%)	Definition
Q1	Numerical accuracy	40	Precision of reported audiometric thresholds and other quantitative test values; deviations >10 dB from the reference or transposed values are flagged as inaccurate.
Q1	Test coverage	30	Inclusion of all relevant audiometric tests presented in the case (eg, pure-tone audiometry, speech testing, immittance, otoacoustic emissions, and auditory brainstem response).
Q1	Description accuracy	20	Correct verbal interpretation of audiometric configuration, symmetry, and severity descriptors.
Q1	Hallucination free	10	Absence of fabricated test values, measurements, or findings not present in the case materials.
Q2	Diagnostic accuracy	50	Correct hearing loss type and severity classification consistent with the reference diagnosis.
Q2	Reasoning logic	30	Sound clinical reasoning chain from findings to diagnostic conclusion.
Q2	Evidence support	20	Diagnostic conclusions explicitly anchored in case findings rather than asserted without justification.
Q3	Relevance to diagnosis	40	Recommendations match the diagnostic impression and presenting concerns.
Q3	Clinical feasibility	30	Recommendations practical and implementable within standard audiology scope of practice.
Q3	Completeness	30	Appropriate scope across amplification, medical referral, follow-up, and counseling as the case requires.

a Subdimension scores are reported on a 1‐ to 5-point scale and combined under the listed weights to produce the weighted score that maps to the holistic letter grade in Table 3; per-evaluation subdimension outputs for the full 804-evaluation set are available at the project repository.

### Image Handling and Multimodal Adjudication

Cases included audiograms, tympanograms, otoacoustic emission results, auditory brainstem response waveforms, and other diagnostic test outputs at varying source resolutions; images were presented in their original published form to the 4 evaluated case study models alongside the textual case history.

In the 50-item reliability audit, the automated case study adjudicator (Claude Opus 4.5) and the 2 human auditors received only the textual reference answer and the model response, not the source images; this ensured that automated and human adjudication there operated on identical inputs. The separate expanded Q1 audit reported below was instead image grounded—its 2 reviewers regraded the responses with the diagnostic images available—which allowed it to bound any net effect of text-only adjudication. For traceability of per-evaluation outputs, the automated adjudicator’s input also included the evaluated model identifier; the automated adjudicator was therefore not blinded to model identity, whereas the human audit reviewers were blinded to model identity (see *Human Audit of Automated Adjudication* section). Implications for fabrication detection are discussed in *Adjudicator Visual Access* section.

### Human Audit of Automated Adjudication

To audit the case study automated adjudicator (Claude Opus 4.5) against expert human judgment, a blinded subsample of 50 case study evaluations was independently rescored by 2 PhD-level audiologists. The audit was conducted as a within-design check on the primary end point and is framed as an audit and calibration of the automated adjudicator rather than as a definitive validation; the sample size of 50 reflects clinician-time constraints in the revision window. The 50-item sample was drawn under stratified random selection (target balance across question type, evaluated model, and adjudicator-assigned A, B, C, or D grade strata) from the 804-evaluation set. The realized sample composition was 17 Q1, 16 Q2, and 17 Q3 items distributed across the 4 evaluated models, with the realized A, B, C, or D grade distribution approximately balanced across grades within each Q-type rather than proportional to the underlying 804-evaluation distribution; realized sample sizes per (Q-type×grade) cell are reported in Table S4 in Multimedia Appendix 1.

Both reviewers were doctoral-level clinical audiologists; one (SW) is a study coauthor, and the other was external to the author team. Reviewers worked independently and were blinded to the identity of the evaluated model that produced each response, to the adjudicator’s labels, and to each other’s labels until both had submitted their full set.

Reviewers recorded a letter grade (per Table 3), a binary any-critical-error flag, and the single best-fit critical-error type when flagged (per Table 4), and a 1- to 2-sentence rationale when an error was flagged or a D grade was assigned without a flagged error. Numerical scores for the human audit comparisons are derived from grade midpoints (mapping in section N.3, Table S2 in Multimedia Appendix 1). The first 8 items served as a calibration segment; rubric drift was checked before the main segment proceeded (per-segment κ values in Table S1 in Multimedia Appendix 1).

Initial preconsensus interreviewer agreement on letter grade was 41 of 50 items in exact agreement (82%); on the binary any-critical-error label, 44 of 50 items were in exact agreement (88%). Quadratic-weighted Cohen κ on letter grade preconsensus was 0.85 (95% CI 0.71‐0.94; bootstrap percentile, *B*=2000); unweighted Cohen κ on the binary any-critical-error label was 0.59 (95% CI 0.34‐0.83). When the 2 reviewers’ grade or critical-error labels diverged, the study team resolved flagged items through consensus adjudication using the prespecified rubric and the 6 critical-error definitions in Table 4, applying each disputed item against the rubric anchor examples and arriving at a final grade and critical-error label per item. This resolution step was conducted on 22 (44%) of 50 items: 9 (18%) items where the 2 reviewers disagreed on grade or critical-error flag and 13 (26%) items where the reviewers agreed on the grade but a divergence between reviewers’ labels and the adjudicator’s label warranted reapplication of the rubric to confirm the human label. The resolved labels for these 22 items, together with the 28 reviewer-agreed items, define the “human consensus” labels used in all reliability analyses mentioned in the following sections.

A first-pass technical review of the adjudication-rationale text was performed using AI assistance (Claude Opus 4.7, distinct from the Claude Opus 4.5 adjudicator under audit) to triage items requiring further study-lead attention; final attestation of all 25 rationale-bearing items (the 22 consensus-resolved items plus 3 additional reviewer-agreed critical-error items requiring rationale) was performed by the corresponding author, with 5 rationales revised at the wording level for technical precision (no consensus labels were changed during attestation). See *Disclosure on Generative AI Use* in the *Acknowledgments* section for the full description.

Interrater agreement was quantified using quadratic-weighted Cohen κ for ordinal letter grades and unweighted Cohen κ for the binary critical-error label, with bootstrap 95% CIs (*B*=2000; deterministic seeds in section N.11 in Multimedia Appendix 1). Intraclass correlation coefficient (ICC) (2,1) absolute agreement on grade midpoint–mapped scores is reported in section N.3, Table S2 in Multimedia Appendix 1.

Two prespecified stratified analyses were performed: per-question-type κ (Q1=audiometric numerical interpretation, Q2=differential diagnosis, and Q3=management recommendations) and a reviewer-agreement subset analysis restricted to the 41 items where the 2 reviewers fully agreed preconsensus (testing whether consensus-formation noise accounts for judge vs human disagreement). Both are tabulated in section N.3, Table S1 in Multimedia Appendix 1.

A 12-cell (question type×adjudicator grade) calibration map was applied to all 804 evaluations as a sensitivity check on the primary Q-type ranking, with the calibrated score computed as human_calibrated_score=clip(original_LLM_score−applied_delta, 0, 100); the full map, sparse-cell fallback rule, and reproducible derivation are in section N.4 (Table S4 in Multimedia Appendix 1).

Two items (S22 and S32) were initially marked “no critical error” by both independent reviewers and were reclassified during consensus adjudication; per-item rationales are in section N.5 in Multimedia Appendix 1. Their contribution is included in the headline κ values reported in the *Audit Agreement* section.

The full audit dataset and analysis scripts are released at the project repository [15] (file inventory in section N.11 in Multimedia Appendix 1).

### Expanded Q1 Audit, Within-Task Human Baseline, and Reference Answer Fidelity

Three additional human audit components were performed in the revision window. The audiologist reviewers for these components were study coauthors (the original 50-item reliability audit, in contrast, had paired 1 study coauthor with 1 external auditor) and were blinded to model identity and, where applicable, to the automated adjudicator’s labels. As the reviewers were members of the study team rather than recruited participants, the study’s original ethics determination (not human subjects research) continued to apply.

#### Expanded Q1 Audit (Image Grounded)

Two PhD-level clinical audiologists independently regraded all 80 model Q1 (audiometric interpretation) responses from the audit sample against Tables 3-5, with the diagnostic audiogram images available to the graders, blinded to model identity and to the automated adjudicator’s labels. This expansion provides (1) interreviewer reliability on a larger Q1 sample, (2) adjudicator versus human reliability for the primary failure task type, and (3) an image-grounded human anchor for the Q1 critical-error rate that operates on richer input than the text-only adjudicator, directly testing whether text-only adjudication distorts the rate. The 80 responses were drawn under a stratified scheme enriched for all-4-model-failure cases; all reliability and rate statistics are reweighted to the 268-evaluation Q1 population using the sampling weights.

#### Within-Task Human Baseline

Two PhD-level audiologists independently answered Q1, Q2, and Q3 on 16 case studies under closed-book conditions before any model response grading. The 16 cases were deliberately enriched for model failure to probe whether the failures are genuine; this is therefore a targeted within-task probe, not a representative baseline. Human answers were scored by the identical automated adjudicator pipeline used for the models (same adjudicator model, rubric version, per-case, per-question, reference answer, and prompt), with the adjudicator blinded to whether each answer was human generated or model generated.

#### Reference Answer Fidelity (SA Tier)

A stratified sample of 210 research article–derived SA items (enriched for all-model-failure plus comparison strata) was audited for reference answer fidelity by a doctoral-level audiologist against the question and full source abstract using a 4-level label (accurate, defensible, flawed, and indeterminate) and independently screened by an LLM (Claude Opus 4.8) on the same items. An objective reference-grounding scan was computed over all 1460 SA items, and a grading-fidelity rejudgment quantified automated-grader false negatives.

### Statistical Analysis

Statistical analyses were performed using Python 3.11 with SciPy 1.11 and pingouin 0.5. Descriptive statistics are reported as mean (SD) with 95% CIs. Parametric CIs based on the *t*-distribution are reported; bootstrap verification (10,000 resamples, percentile method) confirmed near-identical intervals (maximum deviation <0.1 points), supporting robustness to distributional assumptions.

For the primary end point (clinical case studies), nonparametric analyses were used given the bounded and nonnormal distribution of rubric-based scores. The Kruskal-Wallis H test examined main effects of model (4 levels) and question type (3 levels) on the continuous 0 to 100 scores. Post hoc pairwise comparisons used Mann-Whitney *U* tests with Bonferroni correction; all reported post hoc *P* values are Bonferroni adjusted. Following methodological recommendations for nonparametric frameworks [16], we report primary effect sizes as rank biserial correlation (r) for pairwise contrasts and eta-squared based on the Kruskal-Wallis H statistic (eta-squared_H) for omnibus tests. We report Cohen *d* as a supplementary descriptive metric for comparability with the broader LLM evaluation literature; concordance between rank biserial *r* and Cohen *d* across all reported contrasts is verified in Multimedia Appendix 1.

Inferential testing is reserved for the primary end point; the secondary end points (objective item accuracy and research article–derived item accuracy) are reported descriptively due to format-specific ceiling effects (notably TF items at ≥99% accuracy (1175/1186 to 1186/1186 correct per model; Table 6) that limit the discriminative value of intermodel contrasts on those tiers.

**Table 6.** Overall accuracy on research article–derived items^a^.

Model	MCQ^b^ (n=529), n (%)	TF^c^ (n=1186), n (%)	SA^d^ (n=1460), n (%)	Aggregate (n=3175), n (%)
Gemini 2.5 Pro	422 (79.80)	1186 (100)	438 (30.0)	2046 (64.40)
Grok 4	413 (78.10)	1186 (100)	394 (27)	1993 (62.8)
Claude Sonnet 4 Thinking	429 (81.10)	1185 (99.9)	333 (22.8)	1947 (61.3)
OpenAI O3	405 (76.60)	1175 (99.1)	349 (23.9)	1929 (60.8)

a TF items exhibited near-ceiling performance across all models (99.1%-100%), contributing limited discriminative power to the aggregate metric. As TF items constitute 37.4% of the research article pool, they inflate aggregate accuracy substantially. The format-specific breakdowns reveal that SA items are the most discriminative component, with accuracy ranging from 22.8% to 30.0%.

b MCQ: multiple-choice question.

c TF: true/false.

d SA: short answer.

Both adjudicators (Gemini 2.5 Pro on objective and research items; Claude Opus 4.5 on case studies) and all item generation calls used temperature 0; evaluated models were queried under each provider’s default API settings. Indirect evidence against substantial stochastic noise (high interreviewer κ; close concordance across 4 independently developed frontier models) is summarized in the section.

Case-level variability was quantified using ICCs (ICC(1,1); one-way random, single measures) [17]. All tests were 2-tailed with an α value of .05.

Observations are nested within cases (67 cases×4 models×3 question types). The case-level intraclass correlation under the one-way random effects model (ICC(1,1)=0.094) and under the mixed effects model with question type as a fixed effect (residual ICC=0.170) both indicate moderate but limited case-level clustering. Three prespecified sensitivity analyses (Table 7; full per-fold statistics in section N.7 in Multimedia Appendix 1) confirmed that the directional ranking Q3>{Q1, Q2} was preserved under mixed effects modeling, case-level nonparametric bootstrap, and leave-one-case-out resampling.

**Table 7.** Sensitivity analyses for the primary question-type effect^a^.

Analysis	Key statistic	Result	Direction preserved?
Mixed effects model	Q3 versus Q1 fixed-effect coefficient	21.85 (SE 1.26, *P*<.001); Q2 versus Q1=−0.10 (SE 1.26, *P*=.94); residual ICC^b^=0.170	Yes
Case-level nonparametric bootstrap	95% CI for Q3-Q1 mean difference (*B*=1000; seed 20260428)	[18.38, 25.53] for Q3-Q1; [18.93, 24.84] for Q3-Q2; Q3>Q1 in 1000/1000	Yes
Leave-one-case-out (67 folds)	Q1 and Q3 mean range across folds	Q1 [67.44, 68.19]; Q3 [89.58, 90.20]; Q3>Q1 in 67/67	Yes

a Per-fold statistics and the bootstrap script are provided in Multimedia Appendix 1.

b ICC: intraclass correlation coefficient.

**Table 8.** Overall accuracy on educational resource items.

Model	MCQ^a^ (%)	TF^b^ (%)	FIB^c^ (%)	Aggregate (%)
Claude 3 Opus	81.5	93.3	79.3	84.5
Claude Sonnet 4	78.1	85.7	73.0	78.0
Claude Sonnet 4 Thinking	79.8	89.4	72.0	79.0
GPT o4-mini	82.0	91.1	61.6	74.4
GPT-4.1	70.2	73.5	67.3	69.8
GPT-4o	60.7	71.9	61.8	65.3
Gemini 2.5 Pro	81.5	77.2	74.9	76.4
DeepSeek R1	78.6	91.0	65.5	76.0

a MCQ: multiple-choice question.

b TF: true/false.

c FIB: fill-in-the-blank.

## Results

### Objective Items: Online Versus Educational Resources

For MCQs drawn from online resources (N=150 per model), nearly all models exceeded 95% accuracy, ranging from 95.3% (n=143; GPT-4.1) to 98.7% (n=148; Claude 3 Opus). Figure 2 contrasts web-style versus textbook MCQ accuracy by model, highlighting the ceiling effect on online items and the wider spread on curated sources. For MCQs from nonweb sources (N=206 per model), accuracy ranged from 35.0% (n=72) to 72.3% (n=149), providing clear separation among models.

On the 3139 educational items, accuracy declined from TF (72%‐93%) to FIB (60%‐79%). Nonweb MCQ, ranging from 35% (72/206) to 72.3% (149/206), exhibited the widest performance spread and the lowest floor among all formats.

### Research Article–Derived Items

We constructed 3175 items from recent peer-reviewed articles (529 MCQ, 1186 TF, and 1460 SA). Aggregate accuracy on MCQ and TF items was comparable to educational items. SAs yielded the lowest accuracies: Gemini 2.5 Pro achieved 30% (438/1460), Grok 4 27% (394/1460), OpenAI O3 23.9% (349/1460), and Claude Sonnet 4 Thinking 22.8% (333/1460). These results indicated that concise evidence extraction and precise factual recall remain open challenges. Intermodel statistical comparisons were reserved for the primary end point (clinical case analysis). Tables 6 and 8 provide descriptive accuracy for model positioning.

### Clinical Case Studies

#### Overall Performance

Across all 804 evaluations, the overall mean score was 75.14 (SD 19.05). The pass rate (grade C or above) was 684 (85.1%) of 804 evaluations, and the critical-error rate was 105 (13.1%) of 804 evaluations (Table 9; grade distribution in Table 10). Within the rubric definition of dangerous recommendation (Table 4), no responses met that definition across any model (Table 11); we discuss the dataset and rubric scope of this finding in the *Clinical and Regulatory Implications* section.

**Table 9.** Overall case study performance metrics.

Metric	Value
Total evaluations (N)	804
Overall score, mean (SD)	75.14 (19.05)
Overall pass rate (≥C; %)	85.1
Critical-error rate (%)	13.1

**Table 10.** Grade distribution.

Grade	Values, n (%)
A	231 (28.7)
B	237 (29.5)
C	216 (26.9)
D	120 (14.9)
F	0 (0)

**Table 11.** Critical-error distribution^a^.

Error type	Values, n (%)
Type misclassification	34 (4.2)
Severity misclassification	41 (5.1)
Fabricated or erroneous test result	33 (4.1)
Laterality confusion	19 (2.4)
Missed red flag	10 (1.2)
Dangerous recommendation	0 (0)

a Individual responses may contain multiple error types; therefore, the sum of error counts (137) exceeds the number of evaluations containing at least one critical error (105/804, 13.1%).

Formal statistical testing confirmed 2 dominant patterns. The Kruskal-Wallis test revealed a highly significant effect of question type on scores (*χ*²_2_=268.4; *P*<.001; *η*²_H_=0.333), representing a large effect, while model differences were nonsignificant (*χ*²_3_=3.7; *P*=.30; *η*²_H_= 0.001). This indicates that question type, not model selection, is the primary determinant of LLM performance in clinical audiology. The ICC for case ID was 0.094 (*F*_66,737_=2.25; *P*<.001), indicating that approximately 9.4% of score variance was attributable to case difficulty, with the remainder driven by question type and residual variation.

#### Model Performance Comparison

Model performance was relatively consistent. The Kruskal-Wallis test revealed no significant differences among models (*χ*²_3_=3.7; *P*=.30; *η*²_H_=0.001), with mean scores ranging from 73.89 (Grok 4; SD 19.32, 95% CI 71.22‐76.56) to 77.66 (Gemini 2.5 Pro; SD 17.91, 95% CI 75.18‐80.14; Table 12). The negligible effect size indicates that current frontier LLMs did not differ significantly in case study performance on clinical audiology tasks. Gemini 2.5 Pro achieved the highest overall performance with an 87.6% (176/201) pass rate and 11.9% (24/201) critical-error rate.

**Table 12.** Model performance comparison on case studies.

Model	Score, mean (SD)	Pass rate (n=201), n (%)	Critical-error rate (n=201), n (%)
Gemini 2.5 Pro	77.66 (17.91)	176 (87.6)	24 (11.9)
Claude Sonnet 4 Thinking	74.59 (19.50)	167 (83.1)	30 (14.9)
OpenAI O3	74.41 (19.32)	172 (85.6)	24 (11.9)
Grok 4	73.89 (19.32)	169 (84.1)	27 (13.4)

#### Performance by Question Type

Performance varied substantially across question types (Kruskal-Wallis *χ*²_2_=268.4; *P*<.001; η²_H_=0.333), representing a large effect. Q3 (recommendations) achieved the highest mean score (mean 89.74, SD 13.92, 95% CI 88.07‐91.41) with a 98.1% (263/268) pass rate and no responses meeting the rubric definition of dangerous recommendation (see *Overall Performance* section). Post hoc Mann-Whitney *U* tests with Bonferroni correction confirmed that Q3 scored significantly higher than both Q1 (audiometric results; mean 67.89, SD 18.47, 95% CI 65.68‐70.10; *U*=11,540; *P*<.001; rank biserial *r*=0.679; Cohen *d*=1.34) and Q2 (diagnosis; mean 67.79, SD 15.33, 95% CI 65.95‐69.63; *U*=9692; *P*<.001; rank biserial *r*=0.730; Cohen *d*=1.50), both representing very large effects. Critically, the difference between Q1 and Q2 was not statistically significant (*U*=33,573; *P*=.57; rank biserial *r*=0.065; Cohen *d*=0.006), with a negligible effect size, indicating that audiometric interpretation and diagnostic reasoning did not differ significantly in difficulty for current LLMs in the present sample. Q1 showed a critical-error rate of 95 (35.4%) of 268 evaluations, while Q2 demonstrated a pass rate of 250 (93.3%) of 268 evaluations with only 10 (3.7%) of 268 critical errors. The juxtaposition of Q2’s high pass rate with a relatively moderate mean score of 67.79 (SD 15.33) indicates that Q2 scores clustered above the passing threshold yet rarely reached the upper grading tiers, consistent with models producing adequate but seldom excellent diagnostic reasoning (Table 13).

**Table 13.** Performance by question type.

Question type	Score, mean	Pass rate (%)	Critical-error rate (%)
Q1—audiometric results	67.89 (18.47)	63.8	35.4
Q2—diagnostic impression	67.79 (15.33)	93.3	3.7
Q3—clinical recommendations	89.74 (13.92)	98.1	0.0

#### Cross-Analysis: Model×Question Type

**Figure 3.**
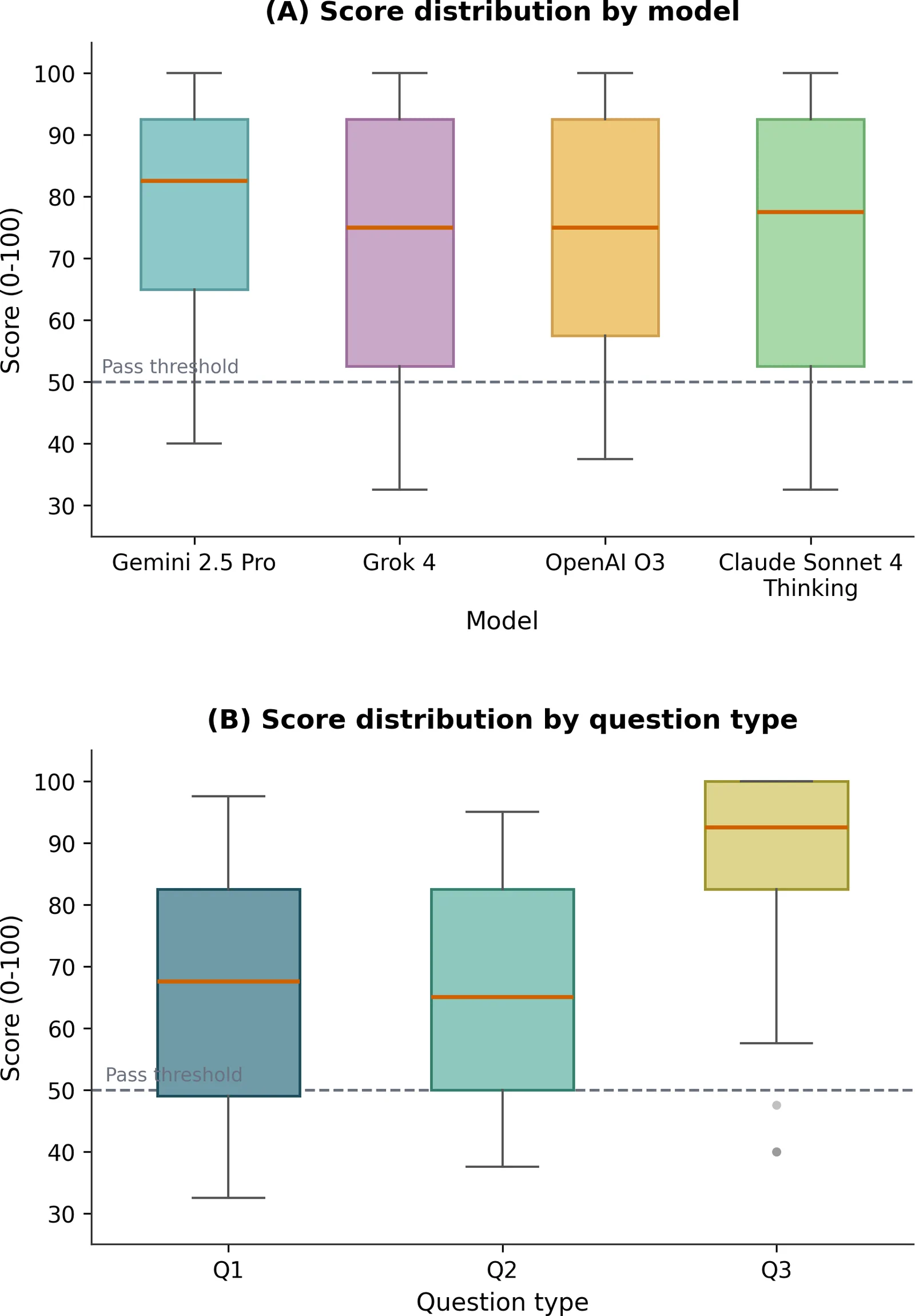
Score distributions: (A) by model and (B) by question type. Dashed line indicates pass threshold (score=50). Box plots show median, IQR, and outliers.

All models excelled at Q3 (recommendations) with mean scores above 88, while all showed lower performance on Q1 (audiometric results) and Q2 (diagnosis). Simple effects analysis with Bonferroni correction (adjusted α=.017 for 3 tests) revealed that model differences were significant only for Q2 (Kruskal-Wallis *χ*²_3_=21.2; *P*<.001; *η*²_H_=0.069, medium effect). Post hoc Mann-Whitney *U* tests with Bonferroni correction (6 pairwise comparisons) confirmed that Gemini 2.5 Pro scored significantly higher on Q2 than each of the other 3 models (all *U*≈3070‐3082; all *P*=.001, all rank biserial *r*=0.37; per-comparison Cohen *d* in Multimedia Appendix 1). The remaining 3 pairwise comparisons were nonsignificant (all *P*>.93). Model differences on Q1 and Q3 were nonsignificant (all *P*>.05). Adjudicator independence considerations are discussed in the section *Adjudicator Independence and Cross-Model Consistency*. Per-model and per-question-type score distributions are shown in Figure 3.

#### Critical-Error Analysis

Critical errors were predominantly concentrated in Q1 responses. Severity misclassification was the most frequent error type at 5.1% (41/804) of all evaluations, followed by type misclassification at 4.2% (34/804). Fabricated or erroneous test result occurred in 4.1% (33/804) of responses.

Within Q1 responses specifically (n=268), critical errors affected 35.4% (n=95) of evaluations (an adjudicator-derived estimate; the expanded human audit indicates that this aggregate rate is consistent with the image-grounded expert estimate, showing no evidence of systematic inflation, although per-item adjudicator labels are noisy; see *Audit Agreement* section). Severity misclassification was the most prevalent error type, followed by fabricated or erroneous test result, type misclassification, and laterality confusion. This concentration of errors in audiometric interpretation underscores that LLM limitations in numerical precision manifest as clinically consequential classification errors.

### Audit Agreement

Interreviewer reliability between the 2 audiologist reviewers on the 50-item audit sample was high overall. Preconsensus exact letter grade match was 41 (82%) of 50 items and preconsensus exact match on the binary any-critical-error label was 44 (88%) of 50 items. Quadratic-weighted Cohen κ for letter grades was 0.85 (95% CI 0.71‐0.94; bootstrap percentile, *B*=2000); unweighted Cohen κ for the binary any-critical-error classification was 0.59 (95% CI 0.34‐0.83). Per-segment κ (calibration vs main) is reported in Table S1 in Multimedia Appendix 1.

Agreement between the case study automated adjudicator (Claude Opus 4.5) and the human consensus was substantially lower. Quadratic-weighted κ for letter grades was 0.31 (95% CI 0.03‐0.55), and unweighted κ for the binary any-critical-error classification was 0.10 (95% CI −0.19 to 0.41). On the binary critical-error classification, the adjudicator showed sensitivity of 25% (3/12), specificity of 84% (32/38), positive predictive value of 33% (3/9), and negative predictive value of 78% (32/41), indicating underdetection of critical errors with modest false-positive flagging in this pooled 50-item sample (the Q1-specific per-item error direction, which is the relevant one for the audiometric-interpretation headline, is characterized on the larger expanded 80-item audit below and reconciled with this pooled figure there). The prevalence-adjusted and bias-adjusted κ (PABAK) [18] was 0.40, the prevalence index was 0.58, the bias index was 0.06, and the maximum κ attainable given the observed marginals was 0.82; together with the raw binary agreement of 70% (35/50), these indices indicate that the unweighted κ of 0.10 reflects the low critical-error prevalence in the subsample 24% (12/50) rather than chance-level agreement, although they do not rescue per-item critical-error detection.

Per-question-type stratification revealed strong heterogeneity in adjudicator-human grade agreement (Table 14; full per-stratum and consensus-rule tables in section N.3 in Multimedia Appendix 1). Quadratic-weighted Cohen κ was 0.57 (95% CI 0.16-0.83) for Q2 and 0.52 (95% CI 0.19-0.76) for Q3, both moderate, but −0.14 (95% CI −0.56 to 0.32) for Q1. The Q1 interval is wide and includes zero: on the 17 Q1 audit items, the point estimate is not statistically distinguishable from chance agreement, and we therefore do not interpret it as evidence of systematically worse-than-chance performance. Two features account for the fragility of the Q1 estimate. First, the audited grades are concentrated in few categories (consensus Q1 grades: B=9, D=7, and C=1), so the marginal variance by which weighted κ is normalized is small, the well-described condition under which κ is paradoxically low despite nontrivial raw agreement [19-21]. Second, leave-one-item-out resampling moved the Q1 κ across the range (−0.25,−0.05), confirming instability at this sample size. Complementary agreement metrics that are not normalized by marginal variance were 18% (3/17) exact and 59% (10/17) within one grade for Q1, versus 50% (8/16) exact and 88% (14/16) within one grade for Q2, and 47% (8/17) exact and 82% (14/17) within one grade for Q3; per-item Q1 grade agreement is therefore genuinely poor but is more faithfully described by these figures than by a single unstable κ.

**Table 14.** Audit agreement summary^a^.

Comparison	N	Grade QW κ^b^ (95% CI)	Any CE κ^c^ (95% CI)	Exact/within-1 grade^d^ (%)
Interreviewer (R88 vs R33), preconsensus	50	0.85 (0.71 to 0.94)	0.59 (0.34 to 0.83)	82/96
Adjudicator versus human consensus (overall)	50	0.31 (0.03 to 0.55)	0.10 (−0.19 to 0.41)	38/76
Adjudicator versus consensus, Q1	17	−0.14 (−0.56 to 0.32)	—^e^	18/59
Adjudicator versus consensus, Q2	16	0.57 (0.16 to 0.83)	—	50/88
Adjudicator versus consensus, Q3	17	0.52 (0.19 to 0.76)	—	47/82

a Bootstrap percentile 95% CIs (*B*=2000). The Q1 grade κ CI includes zero. For the binary any-critical-error label (overall row), raw agreement was 70%, PABAK was 0.40, prevalence index was 0.58, and maximum attainable κ was 0.82 [18].

b Quadratic-weighted Cohen κ for ordinal letter grades.

c Unweighted Cohen κ for the binary any-critical-error label.

d Descriptive agreement percentages not normalized by marginal variance.

e Not applicable.

False positives (n=6) and false negatives (n=9) were dominated by errors involving categorical labeling (laterality, type, and missed red flag) and fabricated test results, respectively; per-item error labels are in the released audit dataset (Section N.11 in Multimedia Appendix 1).

The direction of the adjudicator’s per-item error was characterized on the expanded 80-item Q1 audit, where the adjudicator overflagged critical errors relative to image-grounded human judgment (sensitivity: 19/26, 73%; positive predictive value: 19/53, 36%; 34 overflags versus 7 underdetections). At the population level, the adjudicator’s reweighted Q1 critical-error rate (36.2%) was consistent with the image-grounded human estimate (28%‐34% across the 2 reviewers and label-combination rules), showing no systematic inflation. We withdraw the previous description of the 35.4% (95/268) as a conservative lower bound; per-item adjudicator labels are not treated as reliable, and the capability claim rests on evidence that does not depend on the adjudicator’s per-item reliability (the within-pipeline human baseline, deterministic judge-independent natural language inference (NLI) rescoring, and cross-model replication).

A post-stratified calibration sensitivity analysis applied the 50-item human consensus labels back to the full 804-evaluation set under a 12-cell (question type×adjudicator grade) calibration map. Under this human-calibrated mapping, the Q-type ranking was preserved with Q3 highest (88.4, SD 11.5 calibrated vs 89.7, SD 13.9 uncalibrated), Q1 lowest (65.2, SD 9.1 vs 67.9, SD 18.5), and Q2 intermediate (74.0, SD 9.9 vs 67.8, SD 15.3). The primary Q3>{Q1, Q2} dissociation reported in the main analysis is therefore robust to human-anchored recalibration; the most material shift under calibration is upward movement of Q2 relative to Q1, consistent with the audit-level finding that the adjudicator scored Q2 more harshly than human consensus did. Q-type means under calibration are reported in Table 15.

**Table 15.** Question-type means under original LLM^a^-judge adjudication and human-calibrated sensitivity mapping.^b^

Question type	N	Adjudicator (original)	Human calibrated (sensitivity)
Q1—Audiometric results	268	67.89 (18.47)	65.19 (9.09)
Q2—Diagnostic impression	268	67.79 (15.33)	74.05 (9.90)
Q3—Clinical recommendations	268	89.74 (13.92)	88.38 (11.51)

a LLM: large language model.

b Q3>{Q1, Q2} ranking preserved under human-calibrated mapping. Per-cell calibration deltas and calibrated Q-type means are reported in Multimedia Appendix 1.

Per-item adjudicator labels on Q1 should be interpreted with caution; the aggregate Q-type ranking is preserved under post-stratified human calibration (section N.4, Table S5 in Multimedia Appendix 1). A sensitivity analysis confirmed this ranking is preserved under 3 handling rules for subthreshold calibration cells (the question-type fallback, dropping sparse cells, and a question-type-mean-only map), so the calibration conclusion does not depend on the single sparse cell (one Q2 grade A cell, n=1; section N.4 in Multimedia Appendix 1).

### Multipass Stability

#### Overview

To verify that the Q1 failure mode is stable rather than a single-pass sampling artifact, each case study model was rerun 10 times per case question, and every response was scored against the frozen reference by a deterministic NLI classifier (section N.14 in Multimedia Appendix 1). Aggregate label rates were stable across the 10 passes (Q1 contradiction vs reference: 202/680, 29.7%, SD 3.1 percentage points, range 17/68, 25% to 24/68, 35.3%; Q3: 19/680, 2.8%, SD 1.9 percentage points), and 71% (48/68) of Q1 model case items shared the same label in at least 8 of 10 passes. As the scorer is deterministic, all pass-to-pass variation reflects model generation rather than scorer noise; the contradiction rate indicates stability and is not a reestimate of the 35.4% (95/268) rubric critical-error rate.

#### Expanded Q1 Audit (n=80, Image Grounded)

Two PhD audiologists regraded all 80 model Q1 responses with the diagnostic images available. Interreviewer reliability was high and stable (grade quadratic-weighted κ=0.78, 95% CI 0.68‐0.85; any-critical-error κ=0.91, 95% CI 0.80‐1.00; exact grade agreement: 52/80, 65%, within one grade: 79/80, 99%; the 2 reviewers agreed on the critical-error label for 77/80, 96%, of items). On this larger Q1 sample, the automated adjudicator’s per-item agreement with human judgment was low (grade quadratic-weighted κ=0.05, 95% CI −0.06 to 0.16), superseding the unstable sample size of 17 estimate of −0.14 from the original audit. The direction of the adjudicator’s per-item error was overflagging rather than underdetection: critical-error sensitivity of 73% (19/26, 95% CI 55‐89), specificity of 37% (20/54, 95% CI 25‐51), and positive predictive value of 36% (19/53, 95% CI 24‐49); the adjudicator flagged 34 items as critical errors that the human reviewers did not, versus 7 it missed. This overflagging direction differs from the sensitivity of 25% (3/12) reported for the original 50-item audit; the 2 are reconciled by sample composition rather than treated as conflicting—the earlier 25% (3/12) was an overall figure pooled across question types and rested on only 12 critical-error–positive items (3/12), too few to support a directional Q1 claim, whereas the expanded audit provides the more reliable Q1-specific estimate. Despite the per-item noise, the adjudicator’s aggregate Q1 critical-error rate reweighted to the 268-evaluation population (36.2%) was consistent with the image-grounded human estimate (the 2 reviewers gave 28.3% and 33.6%; a range of 28%‐34% across rules for combining their labels); the human estimates were close to, although slightly below, the adjudicator-derived rate. As the human graders had the audiogram images and the adjudicator did not, this similarity indicates that text-only adjudication does not materially distort the aggregate Q1 critical-error rate.

#### Within-Task Human Baseline (n=16 Enriched Cases)

Two audiologists answered the benchmark under closed-book conditions, before any model response grading; their answers were scored by the identical adjudicator pipeline used for the models. On the shared cases the human Q1 critical-error rate was 3.1% (1/32; Wilson 95% CI 0.6%‐15.7%) versus 81.2% for the models (52/64; 95% CI 70.0%‐88.9%), with nonoverlapping intervals, and on the 11 cases where all 4 models produced a Q1 critical error, the 2 audiologists made zero critical errors (0/22). The human Q1 mean score was 68.3 (SD 9.9, bootstrap 95% CI 64.8‐72.3) and exceeded model means (Claude Sonnet 4 Thinking: 51.4, SD 8.1, to Grok 4: 60.2, SD 18.6). On Q3 both humans (mean 93.4, SD 10.4, 95% CI 89.1‐96.7) and models (Grok 4: mean 84.5, SD 12.9, to Claude Sonnet 4 Thinking: mean 87.3, SD 12.3) were strong, and all 4 models produced 0% dangerous recommendations even on these deliberately hard cases. This indicative within-task probe (enriched, n=16) demonstrates that competent human audiologists answer the same Q1 items with near-zero critical errors, so the model failures reflect a genuine capability limitation rather than unfair or unanswerable questions; it is not juxtaposed with the 35.4% (95/268) population rate. As it is a within-pipeline human versus model comparison, this contrast does not depend on the adjudicator being well calibrated.

## Discussion

### Principal Findings

Our principal finding is a striking capability dissociation in clinical case analysis: LLMs demonstrate strong recommendation generation (Q3 mean 89.74, SD 13.92; no responses meeting the rubric definition of dangerous recommendation) but exhibit significant limitations in both audiometric interpretation (Q1 mean 67.89, SD 18.47; critical-error rate: 95/268, 35.4%) and diagnostic reasoning (Q2 mean 67.79, SD 15.33; critical-error rate: 10/268, 3.7%). Under the original automated adjudication, Q1 and Q2 did not differ significantly with a negligible effect size (see *Performance by Question Type* section), suggesting a fundamental capability boundary between analytical tasks (Q1 and Q2) and synthesis tasks (Q3), rather than a gradual decline across task types. Under human-calibrated sensitivity mapping (Table 15), Q2 shifted upward (mean 74.05, SD 9.90, vs mean 65.19, SD 9.09 for Q1) but remained below Q3 (mean 88.38, SD 11.51); the most robust conclusion is therefore Q3 superiority over both analytical task types. This dissociation is the dominant finding; 3 supporting observations contextualize it. First, web-style MCQ ceiling effects (>95% accuracy; 143/150 to 148/150 correct per model) confirm that examination-style benchmarks are insufficient for evaluating clinical readiness. Second, SA prompts (best: 438/1460, 30%) reveal additional limitations in precise factual extraction. Third, model selection had negligible impact (*η*²_H_=0.001), indicating these are shared frontier LLM limitations rather than model specific.

All 3 prespecified hypotheses are supported: H1 (web-style MCQ ceiling, all 8 evaluated models exceeded 95% accuracy on web-sourced MCQ; 143/150 to 148/150 correct per model); H2 (research article SA accuracy spanned: 333/1460, 22.8%, to 438/1460, 30% and the case study Q1-Q3 dissociation, both providing discriminative information beyond format-saturated items); and H3 (*η*²_H_=0.333 for question type vs 0.001 for model on the case study tier, with the Q1-Q3 dissociation independently replicated within every evaluated model).

### Audit Findings and Shared Q1 Failure

The blinded audit of the case study automated adjudicator (Claude Opus 4.5) against PhD-level audiologist consensus on a 50-item subsample yielded interpretable findings. Interreviewer reliability between the 2 audiologists was high and tightly estimated (grade quadratic-weighted κ=0.85, 95% CI 0.71-0.94; any-critical-error κ=0.59), establishing a stable consensus baseline. Adjudicator versus consensus agreement was lower overall (grade quadratic-weighted κ=0.31, 95% CI 0.03-0.55) and concentrated its disagreement on Q1. We are explicit about what the Q1 audit does and does not support. The adjudicator’s per-item Q1 labels are not interchangeable with expert consensus (exact grade agreement was 3/17, 18%, in the original 17-item Q1 stratum; in the expanded 80-item image-grounded Q1 audit, grade quadratic-weighted κ was 0.05, critical-error sensitivity was 19/26, 73%, and positive predictive value was 19/53, 36%), and we accordingly do not treat per-item Q1 adjudicator labels as reliable. At the same time, the much-cited Q1 quadratic-weighted κ of −0.14 should not be read as systematically worse-than-chance agreement: its 95% CI (−0.56 to 0.32) includes zero, the estimate is destabilized by concentrated grade marginals and a sample of 17 (single items move it by up to 0.20), and the analogous binary κ of 0.10 is depressed by low critical-error prevalence (PABAK=0.40). On the expanded 80-item image-grounded audit, the adjudicator’s per-item errors were in the direction of overflagging rather than underdetection (sensitivity: 19/26, 73%, and positive predictive value: 19/53, 36%); nonetheless, its population-reweighted Q1 critical-error rate (36.2%) was consistent with the image-grounded human estimate (28%‐34%), so the 35.4% (95/268) headline shows no evidence of systematic inflation, although we no longer characterize it as a one-sided lower bound. The substantive Q1 conclusion does not rest on this adjudicator. It is corroborated by (1) a deterministic, judge-independent NLI rescoring of 10 inference passes, in which the Q1 contradiction versus reference rate was stable across passes (202/680, 29.7%, SD 3.1 percentage points, range 17/68, 25% to 24/68, 35.3%) and far exceeded Q3 (19/680, 2.8%) within every evaluated model; (2) replication of the Q3 > Q1 dissociation within each of the 4 independently developed models (Table 16); (3) subdimension localization to numerical accuracy specifically (section N.6 in Multimedia Appendix 1); and (4) preservation of the Q3>{Q1, Q2} ranking under poststratified human calibration (Table 15). We therefore retain the 35.4% (95/268) as an explicitly adjudicator-derived estimate, bracket it with the human-calibrated sensitivity analysis, and rest the capability claim on the judge-independent evidence.

**Table 16.** Scores by model and question type^a^.

Model	Q1 (results), mean (SD)	Q2 (diagnosis), mean (SD)	Q3 (recommendations), mean (SD)
Gemini 2.5 Pro	69.85 (19.00)	74.90 (14.48)	88.25 (14.78)
Grok 4	65.51 (18.05)	65.52 (15.04)	90.63 (12.33)
OpenAI O3	69.72 (18.27)	65.19 (14.78)	88.32 (16.55)
Claude Sonnet 4 Thinking	66.47 (18.56)	65.54 (15.10)	91.75 (11.42)

a All case study scores in this table were assigned by Claude Opus 4.5; Gemini 2.5 Pro served as adjudicator only for the objective item and research article–derived item tiers (see *Models and Inference Protocol* section).

An independent strict-rubric rescoring of the same 50-item subsample by a third PhD-level audiologist (section N.3 in Multimedia Appendix 1), blinded to all prior labels and applying a literal fabrication interpretation, flagged a critical error in 56% (28/50) of the subsample versus 18% (9/50) for each primary reviewer, illustrating that the absolute critical-error rate is strongly dependent on rubric strictness; the 2 primary reviewers, the 2 additional image-grounded reviewers in the expanded Q1 audit, and the automated adjudicator all cluster near one-third under the operational rubric, while a maximally strict literal fabrication interpretation moves the rate higher. The same comparison bounds the reliability of any absolute critical-error rate: agreement between this third reviewer and the primary reviewers was low (pairwise grade quadratic-weighted κ=0.13‐0.26; 3-way Fleiss κ=0.22), indicating that absolute critical-error labeling is strongly rubric-interpretation–dependent even among expert humans. The high primary interreviewer agreement (κ=0.85) should therefore be read as agreement between 2 reviewers who shared a rubric interpretation; what is robust across all reviewers and the automated adjudicator is the directional finding, which the stricter scoring preserved.

Adjudicator deviations concentrate at the score extremes (overgrading Q1 top-bucket responses and overpenalizing the D-bucket on Q1 and Q3, with mid-range Q3 cells well calibrated; per-cell deltas in section N.4, Table S4 in Multimedia Appendix 1)—a pattern consistent with the broader LLM-as-a-judge literature in which automated rubric application is most accurate in the modal score range. Despite these per-item disagreements, the aggregate Q-type ranking (Q3>{Q1, Q2}) is preserved under post-stratified human calibration. We retain the automated-adjudicator scores as the primary headline numbers and report human-calibrated values as a sensitivity analysis (Multimedia Appendix 1).

A case-by-case analysis provides direct evidence that the Q1 limitations are not random and not model specific. Across 67 cases, 11 (16%) Q1 cases elicited grade D from all 4 evaluated models simultaneously (failure defined as grade D, which encompasses both critical-error-capped and noncapped D grades; see section N.8 in Multimedia Appendix 1), compared with 1 Q2 case and 0 Q3 cases; under independence of model failures within a case, the expected count of all-4-fail Q1 cases is 1.15, so the observed 11 is approximately 10 times this expectation. Subdimension analysis (section N.6 in Multimedia Appendix 1) localizes the failure further: Q1 numerical accuracy is uniformly the lowest subdimension across all 4 models, while test coverage is uniformly the highest—current frontier multimodal LLMs can describe which audiometric tests were performed but misstate the specific numerical values.

This concentration is a predictable consequence of the scoring architecture rather than evidence that the Q1 items are unfair. Numerical accuracy carries the largest Q1 subdimension weight (40%; Table 5), whereas evidence support, the lowest-scoring Q2 subdimension, carries the smallest Q2 weight (20%) and is offset by diagnostic accuracy (50%); and, decisively, a mis-stated audiometric value triggers a critical-error cap (severity or type misclassification or a fabricated or erroneous test result) that fixes the grade at D irrespective of the other subdimensions, whereas weak evidence support is not a capped error type. In the data, 95 of 97 Q1 grade D evaluations (98%) carry a critical-error cap versus 10 of 18 (56%) on Q2, so Q1 failures cooccur case wise on the same difficult audiograms, while Q2’s weaker subdimension is diffuse. This is why Q2, despite a lower mean evidence support subdimension, does not show comparable all-model clustering.

### Adjudicator Independence and Cross-Model Consistency

On the primary case study end point, Claude Opus 4.5 (the case study adjudicator) is external to the 4 evaluated case study models, so the triple-role concern that applies to Gemini 2.5 Pro on the secondary tiers (item generation, automated adjudication, and as an evaluated model) does not apply to the primary end point.

Within the case study tier, Gemini 2.5 Pro scored higher than the other 3 evaluated models on Q2 (Gemini 74.90 vs Grok 4 65.52, OpenAI O3 65.19, and Claude Sonnet 4 Thinking 65.54). As these scores were assigned by Claude Opus 4.5 rather than by Gemini 2.5 Pro itself, this differential is not interpretable as self-preference. We retain the descriptive contrast as consistent with a possible cross-model performance difference on diagnostic reasoning, paralleling Gemini 2.5 Pro’s stronger performance on the educational resource items (Table 8). However, because the automated adjudicator was not blinded to model identity (see *Image Handling and Multimodal Adjudication* section) and because Gemini 2.5 Pro also generated the textual reference answer drafts (see following paragraph), the absolute Q2 model ranking should be interpreted with caution.

Although the case study adjudicator (Claude Opus 4.5) is functionally independent of all 4 evaluated models, Gemini 2.5 Pro retains a residual dual role on the primary end point as both reference answer generator and evaluated case study model. Despite the protective design features described in the *Methods* section (no-hallucination extraction constraint; PhD-level reviewer attestation; frozen reference text; adjudication by Claude Opus 4.5), we cannot fully exclude that Gemini-generated reference text shares stylistic conventions with Gemini-generated case study responses, conferring a reference familiarity advantage independent of clinical reasoning quality. Q2 was the only Q-type on which Gemini 2.5 Pro showed a model-specific advantage; the principal Q-type ranking (Q3>{Q1, Q2}) does not depend on this differential, but the absolute Q2 model ranking should be interpreted with this residual concern in mind. Cross-validation against an independent reference generation pipeline is an appropriate follow-up.

Self-preference does remain a relevant concern for the secondary tiers, where Gemini 2.5 Pro served as both adjudicator and evaluated model. This is documented in section N.13.3 in Multimedia Appendix 1, and we have deliberately positioned the case study tier (where this concern does not apply) as the primary end point.

The dominant finding—that task type, not model selection, determines case study performance (rank biserial *r*=0.679 for Q1 vs Q3 and *r*=0.730 for Q2 vs Q3, both representing very large effects; eta-squared_H=0.333 for the question-type omnibus vs eta-squared_H=0.001 for the model omnibus)—is robust to any residual bias because the Q1 to Q3 dissociation is replicated independently within every evaluated model: every model scores above 88 on Q3 and below 70 on Q1 (Table 16). This pattern would persist regardless of systematic adjudicator inflation or deflation.

The weakness in audiometric data interpretation reflects well-documented limitations in LLM numerical precision. Research on mathematical reasoning benchmarks has demonstrated significant performance degradation when numerical values are perturbed [22]. In audiology, where threshold values must be accurately reported within 5 dB clinical precision, this numerical fragility manifests as high critical-error rates. Note that the operational adjudicator applied a wider +10 dB or −10 dB tolerance for flagging numerical inaccuracy in Q1, so that smaller but clinically meaningful 5 to 10 dB deviations are not individually flagged; this tolerance is one source that, in isolation, would tend to lower the Q1 critical-error rate, but it operates in the opposite direction to the adjudicator’s overall per-item overflagging on Q1 (audit agreement), and the net effect is approximately neutral, as the image-grounded human estimate (28%‐34%) was close to, but slightly below, the adjudicator-derived 36.2%, rather than lying systematically above it. The 35.4% (95/268) overall critical-error rate on Q1 indicates that approximately one-third of audiometric interpretations contained clinically significant errors. The 4.1% (33/804) fabricated test result rate observed in our study (Table 11) is consistent with recent evidence that frontier LLMs remain vulnerable to generating fabricated clinical details during decision support [23]. Notably, these errors occurred despite models receiving the original audiogram images as direct visual input, suggesting that current multimodal LLMs have difficulty accurately extracting numerical threshold values from audiogram graphics, further reinforcing the need for clinician oversight.

### Clinical and Regulatory Implications

The 98.1% (263/268) pass rate and the absence of any responses meeting the rubric definition of dangerous recommendation on Q3 support consideration of LLM-assisted tools for generating draft clinical reports, counseling-point summaries, and rehabilitation recommendations pending clinician review. Conversely, the 35.4% (95/268) Q1 critical-error rate forbids autonomous LLM interpretation of audiometric findings; we advocate a hybrid implementation in which LLMs augment rather than replace clinical judgment, deployed for tasks aligned with demonstrated strengths (recommendations, synthesis, and template generation) while audiologists retain exclusive responsibility for audiometric data verification, diagnostic formulation, and final clinical decisions [24,25].

To assess whether the 0% dangerous recommendation rate reflects an insufficiently complex dataset, we draw on the within-task baseline rather than on a separate complexity-coding exercise. The 67 cases were drawn from established audiology casebooks that span presentations explicitly requiring cautious management—asymmetric and unilateral losses, mixed and conductive components, and presentations carrying red flag or urgent referral indications—so the dataset repeatedly affords the opportunity for a contraindicated recommendation. The more direct evidence is provided by the within-task baseline: on the 16 deliberately failure-enriched cases, all 4 models failed Q1 at 81.2% (52/64) yet still produced 0% dangerous recommendations on Q3. The 0% rate therefore persists precisely where the models are demonstrably struggling on the same cases, which is most consistent with the models avoiding contraindicated advice rather than with an absence of contraindication scenarios. We nonetheless interpret the 0% as bounded by this dataset and rubric, and we did not perform a formal, independently verified per-case complexity audit, which we identify as future work.

Pairing an LLM-assisted draft with clinician review does not automatically improve on human-only or AI-only performance. Goh et al [26] reported that physicians using a commercial LLM in diagnostic reasoning did not consistently outperform the LLM operating without physician input. Bergenholtz et al [27] showed that high-performing learners with prior domain knowledge can experience cognitive-load amplification when assessing voluminous generative AI output under time pressure, leading to performance decline relative to a no-AI baseline. The Q1 fabricated test result failure mode (Table 11) maps directly into this hazard: a fabricated audiogram threshold embedded in an otherwise coherent narrative is precisely the failure least likely to be caught by a time-pressured clinician reviewing an LLM draft.

Automation bias is a well-documented hazard in clinical decision support. The structural pattern in our audit calibration map, large automated-adjudicator deviations at score extremes, indicates that automated rubric-based scoring is least reliable at the very high and very low ends, where polished or strongly scored model outputs may still contain clinically consequential errors. Passive verification workflows that rely on automated scoring as a proxy for clinical safety are therefore poorly calibrated to the failure modes documented here. Recent commentary [28] further cautions against uncritical adoption of AI tools in domains where they may displace established practice without commensurate gains in patient outcomes; LLM-assisted audiology tools should be evaluated against the existing standard of care, not against an absence-of-tool baseline.

Three operational implications are as follows: (1) structured verification at decision points: any LLM-assisted tool that generates audiometric interpretations should require explicit clinician verification of every numerical finding (thresholds, configuration, and laterality) before the interpretation is accepted into the patient record, with the verification step actively elicited rather than passively offered; (2) failure mode awareness: clinicians using LLM-assisted tools in audiology should be specifically trained on the fabrication and severity misclassification failure modes documented here because these failures are difficult to detect by surface review alone; and (3) continuous local evaluation: deployments should incorporate ongoing evaluation against local case archives to detect drift in failure rates as models are updated because model behavior on audiology-specific tasks is not guaranteed by general medical licensing performance.

These results characterize capability boundaries on educationally derived materials rather than safety thresholds on real patients; the 35.4% (95/268) Q1 critical-error rate observed across all 4 evaluated models, together with the shared concentration of these errors across 4 independently developed models, indicates that autonomous LLM interpretation of audiometric findings is not currently consistent with safe practice and that switching among current frontier models is unlikely to resolve the issue.

### Comparison With Prior Work

The 85.1% (684/804) overall pass rate exceeds the 75% accuracy reported in Taiwan audiologist qualification examination studies [12], which found performance ranging from 58% on electrophysiological audiology to 88% on basic auditory science. In broader medical contexts, the performance aligns with early ChatGPT performance approaching the USMLE passing threshold [29], while subsequent GPT-4 variants have achieved substantially higher accuracy [2]. Our 3-tier evaluation framework (combining objective items, literature-derived items, and rubric-graded clinical cases) parallels recent holistic approaches, such as HealthBench [8] and MedHELM [9], which similarly move beyond examination-style benchmarks to assess clinical reasoning quality. The finding that LLMs excel at recommendation generation while struggling with numerical precision mirrors patterns observed in other technical specialties; for instance, systematic reviews in ophthalmology [10] have similarly documented strong knowledge retrieval coupled with weaker performance on tasks requiring precise quantitative interpretation. Our use of distinct adjudicators for primary and secondary end points, including transparent documentation of the case study adjudicator’s identity in the present text and a corresponding correction relative to an earlier description of the paper, aligns with emerging best practices for reporting LLM-as-judge designs [30-32]. The blinded human audit of the case study adjudicator (described in *the Human Audit of Automated Adjudication* section) further responds to recent calls for empirical validation of automated-adjudicator labels against expert clinical consensus.

### Comparison With Dedicated Audiogram Interpretation Systems

The performance pattern we observe on Q1 invites comparison with dedicated, non-LLM systems developed specifically for audiogram interpretation. Margolis et al [33] developed AMTAS for automated pure-tone audiometry and QUALIND for quality assessment of the resulting audiograms; Crowson et al [34] developed AutoAudio, a convolutional neural network for audiogram classification; and Charih et al [35] described data-driven audiogram classification approaches. These systems differ from a multimodal LLM in 3 ways. First, they target a narrower task: thresholds and configuration classification, rather than full clinical reasoning over case histories, diagnoses, and recommendations. Second, they accept structured or semistructured input (digital audiogram data points or stylized images of known format) rather than the heterogeneous multimodal materials presented to a generalist LLM. Third, their output is usually a discrete classification or quality-control flag, not a free-text clinical narrative. These task-scope differences make direct head-to-head benchmarking impractical: a comparison of LLM Q1 grades with a dedicated audiogram classifier would be apples to oranges in input modality and output target.

These task-scope differences position the benchmark as informative for clinicians considering generalist LLMs for audiology workflows rather than as a statement about the upper limit of automated audiogram interpretation, where dedicated, task-specific systems remain stronger.

### Limitations

Several limitations should be considered when interpreting our findings; additional methodological limitations are reported in section N.13 in Multimedia Appendix 1.

#### Sample Frame and Case Complexity

The 67 clinical case studies were drawn from established educational resources and may not capture the full diversity of real-world audiological presentations. Although the 804-evaluation design provides 268 observations per question type, the case-level ICC (0.094) indicates that approximately 9% of score variance is attributable to case-specific characteristics; conclusions about absolute model performance on the broader audiological case population should be drawn with caution. Replication on a larger and more diverse case set, including unpublished or institution-internal cases, is a natural extension that subsequent evaluations should pursue.

#### Scope of the Within-Task Human Baseline

An indicative within-task human baseline was collected on 16 case studies deliberately enriched for model failure (see *Audit Agreement* section); 2 audiologists answered under closed-book conditions and were scored by the same adjudicator pipeline as the models, yielding a Q1 critical-error rate of 3.1% (1/32) versus 81.2% (52/64) for the models on the identical cases (0 of 22 critical errors on the 11 all-model-failure cases). The reviewers were study coauthors, blinded to model identity, and answered each case before any model response grading; external multirater replication is a near-term priority. This probe is enriched and small (n=16), is framed as indicative rather than representative, and is not equated with the 35.4% (95/268) population rate; a larger, representatively sampled human baseline scoring under matched constraints remains identified in future directions.

#### Audit and Curation Provenance

The SA reference fidelity labels rest on a single doctoral-level audiologist’s review with an independent LLM screen (Claude Opus 4.8), rather than a multirater human gold standard. Formal item-level preconsensus review records were not retained for the concurrent 2 expert reviews of the objective and research-derived item pools, precluding retrospective estimation of interrater agreement at that curation stage. As discussed in *Adjudicator Independence and Cross-Model Consistency* section, Gemini 2.5 Pro’s dual role as draft reference answer generator and evaluated model leaves a residual stylistic or reference familiarity concern for the absolute Q2 model ranking, although the principal Q3>{Q1, Q2} finding does not depend on that ranking.

#### Sample-Frame Cultural and Linguistic Bias

The 3 evaluation pools were drawn from English-language audiology training materials published primarily in the United States and Australia, and the rubric inherits clinical assumptions of these settings (severity classification anchors, US and Australian referral pathways for the missed red flag category, and audiologist-led scope-of-practice norms for the Q3 recommendation space; full breakdown in section N.13.7 in Multimedia Appendix 1). Performance on AUDIOLOGYBENCH should not be extrapolated to clinical systems with different audiometric reporting conventions, professional structures, or non-English clinical communication. The narrow sampling frame is consistent with broader concerns about WEIRD (Western, educated, industrialized, rich, and democratic) population assumptions in machine learning evaluation pipelines [36]; cross-jurisdiction and cross-language replication is appropriate follow-up.

#### Data Contamination on the Primary End Point

The 67 case studies were drawn from established audiology textbooks and casebooks published between 2020 and 2023. This window falls entirely within the plausible pretraining horizon of all 4 case study models (whose API snapshots all postdate it), so the materials may have been encountered during pretraining, and a date-stratified contamination test is uninformative here: because no case source postdates the models’ training cutoffs, there is no uncontaminable control stratum against which to contrast performance. We also did not run a verbatim completion or membership inference probe across the evaluated models. We therefore cannot exclude that some portion of absolute case study performance reflects training set familiarity. Three considerations bound the impact on our conclusions. First, the cross-question type and cross-model comparisons that carry our claims are driven by relative differences and are far less sensitive to a uniform contamination floor than absolute levels would be. Second, the failure pattern is the opposite of what memorization predicts: verbatim familiarity with textbook cases would, if anything, raise audiometric-interpretation (Q1) accuracy, yet Q1 is the weakest question type (35.4% (95/268) adjudicator-identified critical errors). Third, the Q1 failures concentrated across all 4 models are not the idiosyncratic pattern that memorization of specific cases would produce. A formal verbatim completion audit across the evaluated models is identified as future work. A further consideration is provided by the within-task human baseline: if memorization of these textbook cases were driving model behavior, the models would be expected to perform well on the familiar cases, yet on the 16 shared cases, the models failed Q1 at 81.2% (52/64) while independent human audiologists, facing the identical materials, did not (critical errors: 1/32, 3.1%). A failure mode absent in humans on the same cases is not explained by model memorization.

#### Single-Pass and Multipass Stability

The primary case study evaluations were single pass under the providers’ default API settings. To characterize stochastic stability, we additionally reran the 4 case study models 10 times per case question and scored every response against the frozen reference with a deterministic NLI classifier (see section *Multipass Stability*; section N.14 in Multimedia Appendix 1). Pass-to-pass label rates were stable (Q1 contradiction versus reference: 202/680, 29.7%, SD 3.1 percentage points; Q3: 19/680, 2.8% and 48/68, 71% of Q1 model case items shared the same label in at least 8 of 10 passes, indicating that the Q1 failure mode is not an artifact of single-pass sampling. As the scorer is deterministic, this contradiction rate indicates stability and is not a reestimate of the 35.4% (95/268) rubric critical-error rate.

#### API Versioning and Reproducibility

Two of the 11 model identifiers in Table 2 (gemini-2.5-pro and deepseek-reasoner) are vendor-provided stable aliases rather than date-pinned snapshots and vendor-controlled silent updates to alias-pointed weights between the August 2025 evaluation window and any future rerun cannot be excluded. Date-pinned snapshots for the remaining 9 identifiers, the frozen August 2025 evaluated model responses, and the November 2025 Claude Opus 4.5 adjudicator outputs jointly support replication of all reported analyses; full rederivation of the headline numbers from the released CSV outputs requires no API access.

#### Adjudicator Visual Access

Both the automated case study adjudicator (Claude Opus 4.5) and the human reviewers in the 50-item audit subsample received only the textual reference answer and the model response; neither inspected the original audiogram or other diagnostic images directly. Detection of fabricated audiometric values therefore depends on textual comparison against the reference and may miss fabrications that would be flagged only by direct visual inspection of the source image. This is a deliberate design choice—it ensures that human and automated adjudication operate on the same input—but it constrains fabrication detection to the granularity of the reference text. Five of the 6 critical-error types (type and severity misclassification, laterality confusion, missed red flag, and dangerous recommendation) are fully determined by the textual reference; only a fabricated test result that is internally plausible yet contradicts the unshown image could escape text-only detection. We tested the net effect of text-only adjudication directly: 2 audiologists regraded the model Q1 responses with the diagnostic images available, and their population-reweighted critical-error estimate (28%‐34%) was close to and slightly below the text-only adjudicator’s (36.2%), bounding any net text-only overpenalty at a few percentage points and indicating that text-only adjudication does not materially distort the aggregate Q1 critical-error rate. The adjudicator’s nonblinding to model identity likewise cannot account for the task-type dissociation, which is reproduced by a deterministic NLI scorer that never receives a model identifier. An image-grounded, model-blinded readjudication is identified in the *Future Directions* section as the way to close both gaps.

#### Compute and Energy-Footprint Accounting

The study did not include compute, energy, or carbon accounting for the 804 case study evaluations or for model inference over the 6314 objective and research-derived items; we identify this as a desirable addition to future benchmark iterations [37].

### Future Directions

Three priority directions emerge from this work. First, multijudge designs incorporating a second independent automated adjudicator alongside larger multireviewer human audit panels with formal prescoring rubric calibration would help disentangle adjudicator-specific effects from genuine model performance differences. Second, a larger, externally recruited, representatively sampled within-task human audiologist baseline on the full case set, beyond the indicative enriched probe reported here, would establish clinically meaningful performance thresholds and contextualize LLM capabilities relative to clinical practice. Third, cross-jurisdiction and cross-language replication would address the cultural-linguistic representativeness limitation noted earlier. Additional directions, including domain-specific fine-tuning, retrieval-augmented generation, multipass stochastic-stability characterization, formal contamination audits, image-resolution effects, and human-AI collaboration dynamics, lie outside the closed-book snapshot scope of this study.

### Conclusions

AUDIOLOGYBENCH evaluates frontier LLM performance on clinical audiology along 3 evaluation tiers (curated knowledge, literature-derived evidence, and multimodal case reasoning). On the primary case study end point, current frontier LLMs (Gemini 2.5 Pro, Grok 4, OpenAI O3, and Claude Sonnet 4 Thinking) demonstrate strong recommendation generation (mean 89.74, SD 13.92, 98.1% (263/268) pass rate within this dataset and rubric, no responses meeting the rubric definition of dangerous recommendation) and substantial limitations in audiometric numerical interpretation (mean 67.89, SD 18.47, 35.4% (95/268) critical-error rate). Under the original automated adjudication, diagnostic reasoning performance did not differ significantly from audiometric interpretation (rank biserial *r*=0.065); under human-calibrated sensitivity mapping (Table 15), Q2 shifted upward but remained below Q3, and Q3 superiority over both analytical task types was preserved. Question type, not model selection, dominated case study performance (eta-squared_H=0.333 vs 0.001), indicating that the observed limitations are shared across current frontier LLMs rather than model specific.

A blinded human audit of the case study automated adjudicator, expanded to 80 image-grounded Q1 regradings by 2 PhD audiologists, showed high interreviewer reliability (grade quadratic-weighted κ=0.78 in the expanded Q1 audit and 0.85 in the original 50-item audit) and confirmed that per-item Q1 adjudicator labels are noisy (overflagging; quadratic-weighted κ=0.05), while the aggregate Q1 critical-error rate was consistent with the image-grounded human estimate (36.2% adjudicator vs 28%‐34% human), showing no evidence of systematic inflation. An indicative within-task human baseline (3.1% (1/32) human vs 81.2% (52/64) model Q1 critical errors on shared cases) and an SA reference fidelity audit (100% key fidelity; corrected best-model score 33.2% (485/1,460) or below) further support that the documented limitations reflect genuine model capability boundaries. The benchmark therefore robustly identifies a task-type capability boundary in current frontier LLMs while providing transparent disclosure of adjudicator-level limitations that constrain per-item interpretation.

## Supplementary material

10.2196/94755 Multimedia Appendix 1 Supplementary materials supporting AUDIOLOGYBENCH, including evaluation-stage prompt documentation, the case study adjudicator output schema, full audit agreement and human calibration tables, subdimension means by question type and model, 3 case-level sensitivity analyses, shared failure tabulation, the full A-F rubric reference, representative item examples, and extended limitations.

## Abbreviations

FIB: fill-in-the-blank
ICC: intraclass correlation coefficient
LLM: large language model
MCQ: multiple-choice question
NLI: natural language inference
PABAK: prevalence-adjusted and bias-adjusted κ
QA: question answering
SA: short answer
TF: true/false
USMLE: United States Medical Licensing Examination
WEIRD: Western, educated, industrialized, rich, and democratic
