# Supplementary material for: Evaluating Large Language Models in Clinical Audiology (AUDIOLOGYBENCH): Benchmark Development and Validation Study
Source: J Med Internet Res. 2026 Sep 11;28:e94755. doi: 10.2196/94755 (PMC13568690; doi:10.2196/94755)
Supplement: Supplementary file 1 [file jmir-v28-e94755-s001.docx]

**Multimedia Appendix**

*Supplementary Materials for AUDIOLOGYBENCH*

Evaluating Large Language Models in Clinical Audiology

This appendix collects the supplementary materials referenced in the main manuscript: prompts used at each evaluation stage, full per-stratum agreement and calibration tables, sub-dimension means by question type and model, sensitivity-analysis results (mixed-effects, case-level bootstrap, leave-one-case-out), shared-failure tabulations, the full A–F rubric reference, and the audit dataset and analysis-script provenance.

**Sections:**

- N.1 Prompts used at each evaluation stage
- N.2 Cases adjudicator output schema
- N.3 Audit-agreement full tables (per-stratum kappa, ICC, alternative consensus rules)
- N.4 Calibration map and Q-type x Model means under human-anchored mapping
- N.5 Critical-error reclassification audit trail (S22, S32)
- N.6 Sub-dimension means by Q-type and model
- N.7 Case-level sensitivity analyses (mixed-effects, bootstrap, LOOCV)
- N.8 Shared-failure tabulation (B3 reviewer comment)
- N.9 Rubric reference table with sub-dimension definitions
- N.10 Cohen's d vs rank-biserial r concordance
- N.11 Audit dataset, analysis script, and provenance
- N.12 Representative item examples
- N.13 Extended limitations
- N.14 Multi-pass stochastic stability of the task-type dissociation
- N.15 Expanded image-grounded Q1 audit: reviewer and adjudicator reliability
- N.16 Within-task human baseline
- N.17 Short-answer reference-answer fidelity audit

# N.1 Prompts Used at Each Evaluation Stage

This section reproduces, verbatim, the full prompt used at each evaluation stage, together with its source inputs, output schema, and key constraints. The case-study adjudicator prompt template (Claude Opus 4.5; system message, user-message template, per-question-type substituted criteria, dimension-score JSON templates, and the deterministic post-adjudication score-computation algorithm) is reproduced in Section N.1.8. The full prompt text for every preserved stage is reproduced verbatim in the subsections below; the corresponding prompt JSON files are also released in the project repository. The corresponding per-evaluation output schema is documented in Section N.2.

## N.1.1 Item generation - Objective MCQ items (`MCQ_items.json`)

Used with Gemini 2.5 Pro at temperature 0 to generate the 356 multiple-choice items in the objective tier from audiology textbook TOC + chapter key points.

Source: audiology textbook TOC array and chapter key-points dictionary.

Output: JSON array of items each containing chapter, cognitive level (Recall vs Application), original key point, question stem, four options, correct answer letter, rationale.

Constraints: ≤ 180 tokens per item; one well-designed item per key point; ~50% Recall / ~50% Application; correct answer roughly evenly distributed across A/B/C/D; no 'All of the above' / 'None of the above' / 'Both A and B' compound options; mutually exclusive parallel-grammar options; randomised correct-option position.

**Full prompt (`MCQ_items.json`), reproduced verbatim:**

```
# ROLE
You are an expert in audiology education and assessment design with extensive experience writing high-stakes multiple-choice items.

# INPUT
You receive two JSON fragments **in this order**:

1. A TOC array – each element has
   { "level": "...", "chapter_number": <int>, "title": "...", ... }
2. A dictionary called `chapters` – keys are `"Chapter 1" … "Chapter 15"`;
   each value is an array of bullet-style key points.

Example (truncated):
[
  { "level": "Chapter", "chapter_number": 1, "title": "Introduction", ... },
  …
]
{
  "Chapter 1": [
    "The WHO uses the ICF for considering a health-related disability.",
    …
  ],
  …
}

Use *only* this content.

# TASK
For **every** key point in `chapters`, write **one** four-option, single-best-answer multiple-choice item that determines whether a learner is ready to function as a competent audiologist.

## STEP 1 – select a cognitive level
• **Recall** – probes memory of a definition, classification, value, standard, or named procedure stated in the key point.
• **Application** – probes clinical reasoning by embedding the key point in a brief case vignette or applied scenario.

Across the full item set, target roughly 50 % Recall and 50 % Application.

## STEP 2 – write the stem
• The stem must be a single, complete, unambiguous question or sentence-completion prompt that can be answered without seeing the options.
• Frame the stem positively wherever possible. If a negative phrasing is unavoidable, capitalise the negative word (e.g. NOT, EXCEPT, LEAST).
• For Application items, prepend a 1–2 sentence vignette (patient age, complaint, relevant test result, listening environment, etc.) drawn ONLY from concepts present in or directly implied by the key point.
• Avoid grammatical or length cues that hint at the correct option (e.g. "a/an" giveaways, conspicuously longer correct answer).

## STEP 3 – write four options (A–D)
• Exactly **one** option must be unambiguously correct and defensible from the key point.
• Each of the three distractors must be plausible to a learner with only superficial knowledge: common misconceptions, adjacent terminology, near-miss numerical values, or procedurally similar but incorrect choices.
• Options must be mutually exclusive, parallel in grammar, and similar in length.
• Do **not** use "All of the above", "None of the above", "Both A and B", or other compound options.
• Randomise the position of the correct answer; across the full set, the correct letter should be roughly evenly distributed among A, B, C, and D.

## STEP 4 – minimal context tag
If the stem would be ambiguous in isolation (e.g. "this test", "the device"), prepend the **shortest possible** context string
`[<Chapter-title> – <Section-title>] ` using TOC data.

# OUTPUT
Return **one valid JSON array** – nothing before or after it.
Follow this schema **exactly** (no comments inside the array):

SCHEMA
{
  "chapter":   <integer>,
  "cognitive": "Recall" | "Application",
  "original":  "<exact key point>",
  "question":  "<stem text>",
  "options": {
    "A": "<string>",
    "B": "<string>",
    "C": "<string>",
    "D": "<string>"
  },
  "answer":    "A" | "B" | "C" | "D",
  "rationale": "<one-sentence justification grounded in the key point>"
}

# FEW-SHOT EXAMPLES – imitate format, style, length
[
  {
    "chapter": 4,
    "cognitive": "Recall",
    "original": "The pure-tone average (PTA) is calculated as the arithmetic mean of hearing thresholds at 500, 1000, and 2000 Hz.",
    "question": "Which frequencies are averaged to obtain the conventional pure-tone average (PTA)?",
    "options": {
      "A": "250, 500, and 1000 Hz",
      "B": "500, 1000, and 2000 Hz",
      "C": "1000, 2000, and 4000 Hz",
      "D": "500, 2000, and 4000 Hz"
    },
    "answer": "B",
    "rationale": "The conventional PTA is the arithmetic mean of hearing thresholds at 500, 1000, and 2000 Hz."
  },
  {
    "chapter": 5,
    "cognitive": "Application",
    "original": "Conductive hearing loss is characterised by an air–bone gap with normal bone-conduction thresholds.",
    "question": "A 32-year-old patient reports unilateral hearing reduction in the right ear following a recent upper-respiratory infection. Audiometry shows right air-conduction thresholds of 45 dB HL across the speech frequencies, while right bone-conduction thresholds remain at 5 dB HL. Which type of hearing loss does this pattern indicate?",
    "options": {
      "A": "Sensorineural hearing loss",
      "B": "Mixed hearing loss",
      "C": "Conductive hearing loss",
      "D": "Auditory neuropathy spectrum disorder"
    },
    "answer": "C",
    "rationale": "An air–bone gap with normal bone-conduction thresholds is the defining audiometric pattern of conductive hearing loss."
  }
]
# END EXAMPLES

Constraints
• **≤ 180 tokens** per item (stop if the limit is reached).
• Produce no duplicate items and no text outside the JSON array.
• Every correct answer and every distractor must be defensible from the key point or from established audiology knowledge consistent with it.
```

## N.1.2 Item generation - Objective TF/FIB items (`TF_FIB_items.json`)

Used with Gemini 2.5 Pro at temperature 0 to generate the 1,112 true/false and 1,671 fill-in-the-blank items in the objective tier.

Source: same audiology textbook TOC and chapter key points.

Item-type selection rule: TF when the statement lacks specialised audiology terminology; FIB when knowledge of terminology / procedures / standards is required.

Constraints: ≤ 120 tokens per item; ~50% of all TF items have answer 'False'; FIB items mask one key term (occasionally up to three); each blank ≤ 2 words; verbatim correct answer.

**Full prompt (`TF_FIB_items.json`), reproduced verbatim:**

```
# ROLE
You are an expert in audiology education and assessment design.

# INPUT
You receive two JSON fragments **in this order**:

1. A TOC array – each element has
   { "level": "...", "chapter_number": <int>, "title": "...", ... }
2. A dictionary called `chapters` – keys are `"Chapter 1" … "Chapter 15"`;
   each value is an array of bullet-style key points.

Example (truncated):
[
  { "level": "Chapter", "chapter_number": 1, "title": "Introduction", ... },
  …
]
{
  "Chapter 1": [
    "The WHO uses the ICF for considering a health-related disability.",
    …
  ],
  …
}

Use *only* this content.

# TASK
For **every** key point in `chapters`, create **one** assessment item that determines whether a learner is ready to function as a competent audiologist.

## STEP 1 – choose item type
• **TF** (True/False)  – when the statement lacks a specialised audiology term.
• **FIB** (Fill-in-the-Blank) – when knowledge of terminology / procedures / standards is needed.

## STEP 2 – write the item
### True/False
• Recast as a single sentence answerable “True” or “False”.
• Make ≈ 50 % of all TF items False (re-phrase if necessary).

### Fill-in-the-Blank
• Rewrite as one clear sentence, then hide **1 key audiology term** (preferred) or ≤ 3 when it markedly improves discrimination.
• Each blank masks ≤ 2 words; most items get exactly one blank.
• The blank must have **one unambiguous correct answer**, verbatim from the key point.
• Expand abbreviations in the visible sentence if that aids clarity; keep the answer unchanged.

### Context
If the item would be ambiguous in isolation, prepend the **minimal** context string
`[<Chapter-title> – <Section-title>] ` using TOC data.

# OUTPUT
Return **one valid JSON array** – nothing before or after it.
Follow this schema **exactly** (no comments inside the array):

SCHEMA
{
  "type":    "TF" | "FIB",
  "chapter": <integer>,
  "original":"<exact key point>",
  "question":"<rewritten assessment item>",
  "answer":  "<string>" | ["<string>", ...]
}

# FEW-SHOT EXAMPLES – imitate format, style, length
[
  {
    "type": "TF",
    "chapter": 1,
    "original": "The WHO uses the ICF for considering a health-related disability.",
    "question": "True or False: The World Health Organization relies on the International Classification of Functioning, Disability and Health (ICF) when describing hearing-related disability.",
    "answer": "True"
  },
  {
    "type": "FIB",
    "chapter": 1,
    "original": "Hearing loss may limit communication activity and impose participation restrictions on everyday activities.",
    "question": "Hearing loss can restrict ________ activity and limit participation in everyday life.",
    "answer": "communication"
  }
]
# END EXAMPLES

Constraints
• **≤ 120 tokens** per item (model should stop if limit is reached).
• Produce no duplicate items and no text outside the JSON array.
```

## N.1.3 Item generation - Research-article-derived items (`research_article_prompts.json`)

A two-step pipeline used with Gemini 2.5 Pro at temperature 0 to convert peer-reviewed audiology paper conclusions into 3,175 exam items (MCQ/TF/SA).

Step 1 (Suitability Screening) determines whether a paper conclusion contains generalizable knowledge appropriate for stand-alone assessment items, returning `suitable: true|false` per paper.

Step 2 (Question Adaptation) generates exactly one well-designed item per suitable conclusion, choosing the most appropriate type (MCQ with four options, TF, or SA) and providing the reference answer. Items must not mention 'study,' 'paper,' 'research,' or 'conclusion'; abbreviations are expanded at first use; numbers/p-values preserved verbatim.

**Step 1 - Suitability Screening (`research_article_prompts.json`), reproduced verbatim:**

You are an expert audiology educator and assessment-design specialist.

INPUT
The user message will contain one or more JSONL lines.
Each line has at least the keys "title" and "conclusion".

TASK
For every input line whose "conclusion" field is non-empty:
1. Evaluate whether the conclusion contains sufficiently generalized knowledge that would be appropriate for educational assessment.
2. Determine if the conclusion can be converted into stand-alone exam questions without requiring knowledge of the specific study context.
3. Provide a clear explanation of your reasoning.

EVALUATION CRITERIA
A conclusion is SUITABLE if it:
- States generalizable principles, relationships, or facts about audiology
- Contains actionable clinical knowledge or established findings
- Can be understood and applied beyond the specific study context
- Provides clear, measurable outcomes or recommendations

A conclusion is NOT SUITABLE if it:
- Only describes study-specific methodology or limitations
- Contains vague or inconclusive statements
- Requires detailed knowledge of the research context to understand
- Lacks clear, testable knowledge components

OUTPUT FORMAT
Write one valid JSON object per line (JSONL) for every conclusion you evaluate, using this key order:
"ID"
"title" - the original title
"conclusion" - the original conclusion text
"suitable" - true or false
"explanation" - detailed reasoning for your decision (2-3 sentences)
"key_concepts" - list of specific testable concepts if suitable, empty array if not suitable

Keep each JSON object on a single physical line (no internal line breaks).

**Step 2 - Question Adaptation, reproduced verbatim:**

```
You are an expert audiology educator and assessment-design specialist.

INPUT
The user message will contain one or more JSONL lines from papers that have been pre-screened as suitable for question generation.
Each line has at least the keys "title" and "conclusion".

TASK
For every input line whose "conclusion" field is non-empty:
1. Treat that conclusion as a stand-alone knowledge statement.
2. ***Create exactly one well-designed exam question*** that can be answered solely from that knowledge.
3. Choose the most appropriate question type for that idea:
   - True/False - label "TF"
   - Multiple-choice with exactly four options (A-D) - label "MCQ"
   - Short-answer that requires a single clear phrase, number, or sentence - label "SA"
   (Quality over quantity; you do NOT need to supply all three types.)

STYLE & STAND-ALONE REQUIREMENTS
- Questions must NOT mention or hint at a "study," "paper," "research," "abstract," or "conclusion."
- Each question stem must include every factual detail needed for a correct answer.
- Expand each abbreviation at first use, followed by the abbreviation in parentheses.
- Paraphrase - do not lift large verbatim chunks. Keep all numbers, statistics, and p-values exactly as written.
- Use plain ASCII; no Markdown.

OUTPUT FORMAT
Write one valid JSON object per line (JSONL) for every question you generate, using this key order:
"source_title"  - the original paper title for reference
"question_text" - full question stem (include options if MCQ)
"answer"        - correct answer (exact phrase, option letter, or list)
"question_type" - "TF", "MCQ", or "SA"

Keep each JSON object on a single physical line (no internal line breaks).
For MCQs, supply four plausible distractors and give the correct option letter in "answer".
```

## N.1.4 Reference-answer extraction for case studies (`audiology_case_study_prompt.json`)

Used with Gemini 2.5 Pro at temperature 0 to generate the three-section reference answer (RESULTS / IMPRESSION-DIAGNOSIS / RECOMMENDATIONS) for each of the 67 case studies. Reference drafts were subsequently reviewed by a doctoral-level audiology reviewer (the corresponding author) for clinical accuracy and alignment with the source case material; reviewer-flagged drafts were corrected before the reference set was frozen.

Output schema: JSON object containing `case_id`, `RESULTS`, `IMPRESSION_DIAGNOSIS`, `RECOMMENDATIONS`. Each section is a prose paragraph ≤ 150 words.

Critical constraints: 'NO HALLUCINATION: Only include information explicitly stated in the document'; 'If tests are described as recommended/pending/unknown, state this explicitly'; 'Do not infer outcomes, diagnoses, or test results that are not directly reported'; 'If critical information is not mentioned, omit rather than assume.'

**Full prompt (`audiology_case_study_prompt.json`), reproduced verbatim:**

\# Audiology Case Study Information Extraction

You are an expert audiologist tasked with extracting clinical information from audiology case studies to create structured ground truth summaries. Read the entire case study carefully and generate a summary with exactly three sections.

\## Output Requirements

- **Format:** Professional prose paragraphs (NO bullet points, numbered lists, or special formatting)
- **Length:** Each section must be ≤150 words
- **Style:** Concise, clinical documentation style with flowing sentences
- **Terminology:** Use precise audiological terminology with numerical values (dB HL, dB SPL, %, Hz, mL, daPa)

\## Critical Rules

1. **NO HALLUCINATION:** Only include information explicitly stated in the document
2. **Pending/Unknown Results:** If tests are described as "recommended," "pending," "unknown," or "not completed," state this explicitly (e.g., "Results were pending at time of report")
3. **Distinguish Clearly:** Separate completed tests from recommended/planned tests
4. **No Assumptions:** Do not infer outcomes, diagnoses, or test results that are not directly reported
5. **Omissions:** If critical information is not mentioned, omit it rather than assuming; do not write "Not reported"

\## Section Definitions

**RESULTS:**
Summarize all completed objective test findings including: pure-tone audiometry (type, degree, configuration), speech audiometry (SRT, WRS with test materials and presentation levels), immittance measures (tympanogram type, acoustic reflexes), hearing aid verification (REM targets, input levels), aided testing outcomes, and any other completed diagnostic tests. Include specific numerical values.

**IMPRESSION/DIAGNOSIS:**
Provide clinical interpretation including: primary diagnosis with relevant history/duration, hearing loss classification, symptom description (tinnitus, vertigo, etc.), notable or unexpected findings, asymmetries or red flags, relevant case history (noise exposure, family history, medical history), and the clinical relationship between test results and reported symptoms.

**RECOMMENDATIONS:**
Summarize clinical recommendations including: amplification decisions (device type, style, features, coupling), medical referrals made or recommended, additional diagnostic testing (completed vs. pending/recommended), tinnitus management, assistive technology (current and planned), patient counseling points, follow-up plans, and treatment outcomes if reported. Clearly indicate which recommendations were implemented versus planned for future.

\## Output Format

Return a JSON object with the following structure:
```json
{
 "case_id": [number],
 "RESULTS": "[prose paragraph ≤150 words]",
 "IMPRESSION_DIAGNOSIS": "[prose paragraph ≤150 words]",
 "RECOMMENDATIONS": "[prose paragraph ≤150 words]"
}
```

\## Example Output Style

RESULTS: "Comprehensive audiometry revealed bilateral symmetrical moderately severe sensorineural hearing loss with a flat configuration. Speech Recognition Thresholds were 75 dB HL (right) and 65 dB HL (left), consistent with pure-tone averages. Word Recognition Scores using recorded NU-6 materials with male talker at 85 dB HL were 50% for the right ear and 66% for the left ear. Aided word recognition at 65 dB SPL showed 40% (right monaural), 66% (left monaural), and 50% (bilateral). Tympanometry revealed Type A tympanograms bilaterally with normal middle ear pressure and compliance. Acoustic reflex thresholds were absent bilaterally from 500 to 4000 Hz. Real-ear measurements confirmed NAL-NL2 targets were achieved within 5 dB at 50, 65, and 80 dB SPL input levels."

## N.1.5 Evaluated-model system prompt for case studies (`audiologist_prompt.json`)

Used identically for all four evaluated case-study models (Gemini 2.5 Pro, Grok 4, OpenAI O3, Claude Sonnet 4 Thinking) on each of the 201 (67 cases x 3 question types) evaluations per model. Each evaluated model received the system prompt, the case record (text), and diagnostic image inputs (audiograms, tympanograms, REM screenshots, etc.).

Persona: 'You are a board-certified clinical audiologist with 15+ years experience in hearing loss, tinnitus, and vestibular disorders.'

Output template: three sections (Results, Impression/diagnosis, Recommendations), each ≤ 150 words. Constraint: 'When citing a data point, reproduce it exactly (e.g., "Right-ear WRS = 50 %")'; 'Present only your final, evidence-based conclusions; do not include any reasoning explanations or chain-of-thought.' Missing-section rule: 'If a section is missing or lacks sufficient information, write "Information not provided."'

**Full prompt specification (`audiologist_prompt.json`), reproduced verbatim:**

```
{
  "role": "system",
  "persona": "You are a board-certified clinical audiologist with 15+ years' experience in hearing loss, tinnitus, and vestibular disorders.",
  "input": {
    "description": "A complete case record will be supplied as a JSONL file.",
    "notes": "Linked or embedded images may include audiograms, tympanograms, REM screenshots, etc."
  },
  "task": [
    "Parse the entire JSONL record and review every linked image.",
    "Based solely on the case information and widely accepted audiological principles, provide concise, professional content for each of the following sections if present: Results, Impression/diagnosis, Recommendations.",
    "Write clearly in graduate-level English."
  ],
  "output_format": {
    "instruction": "Follow exactly the structure below.",
    "template": "**Results:**\n<your answer>\n\n**Impression/diagnosis:**\n<your answer>\n\n**Recommendations:**\n<your answer>",
    "missing_section_rule": "If a section is missing or lacks sufficient information, write \"Information not provided.\""
  },
  "constraints": [
    "Each section ≤ 150 words.",
    "When citing a data point, reproduce it exactly (e.g., \"Right-ear WRS = 50 %\").",
    "Present only your final, evidence-based conclusions.",
    "Do not include any reasoning explanations or chain-of-thought."
  ],
  "begin": true
}
```

## N.1.6 LLM-judge for FIB and SA grading (`prompt_2_grader.json`)

Used with Gemini 2.5 Pro at temperature 0 to grade evaluated-model FIB responses (educational objective tier) and SA responses (research-article-derived tier) against the reference answers.

Grading rules: an answer is correct if any of (i) normalized exact match (lowercased, stripped of leading/trailing punctuation), (ii) verbatim substring (model answer contains the reference verbatim with extra words allowed), (iii) parenthetical acronym difference, (iv) generic filler-noun difference (e.g., 'probe microphone technology' vs 'probe microphone'), or (v) expert-judgment true synonyms in standard audiology usage.

Multiple-choice item adjudication was deterministic exact-match against the reference option letter (no LLM-judge call needed); the LLM-judge above applies to FIB and SA items only.

**Full prompt specification (`prompt_2_grader.json`), reproduced verbatim:**

```
{
  "role": "expert audiology educator",
  "task": {
    "description": "Grade each item by comparing the current answers with Correct Answers in the same folder.",
    "inputs": {
      "model_answers": "The answers produced by the answerer (current answers file)",
      "correct_answers": "The reference answers located in the same folder"
    }
  },
  "grading_criteria": {
    "description": "Treat an answer as correct if ANY of the following conditions is true.",
    "conditions": [
      {
        "id": 1,
        "name": "normalized_exact_match",
        "rule": "After lower-casing and stripping leading/trailing spaces and punctuation, the strings match exactly."
      },
      {
        "id": 2,
        "name": "verbatim_substring",
        "rule": "The model_answer contains the entire correct phrase verbatim, even if extra words appear before or after."
      },
      {
        "id": 3,
        "name": "parenthetical_acronym_difference",
        "rule": "The only difference is a parenthetical acronym/abbreviation.",
        "example": {
          "correct": "Transfer-Appropriate Processing",
          "model": "Transfer-Appropriate Processing (TAP)"
        }
      },
      {
        "id": 4,
        "name": "generic_filler_noun_difference",
        "rule": "The model_answer adds or drops a generic filler noun.",
        "filler_nouns": [
          "test",
          "technique",
          "technology",
          "training",
          "assessment",
          "procedure",
          "approach",
          "method"
        ],
        "example": {
          "equivalent_pair": [
            "probe microphone technology",
            "probe microphone"
          ]
        }
      },
      {
        "id": 5,
        "name": "expert_synonym_judgment",
        "rule": "In your expert judgment the terms are true synonyms in standard audiology usage."
      }
    ]
  },
  "output_format": {
    "per_item_line": {
      "template": "<index>. <mark>  (<type>)",
      "marks": {
        "correct": "✅",
        "incorrect": "❌"
      },
      "incorrect_suffix": {
        "required_when": "mark is ❌",
        "template": " – expected: <correct>, got: <model_answer>"
      },
      "ordering": "Original order of items must be preserved."
    },
    "summary_line": {
      "position": "After the last item line",
      "template": "Score: <correct_count>/<total> (Accuracy = <rounded_percent>%)",
      "accuracy_rule": "Accuracy is a whole-number percentage rounded to the nearest integer."
    }
  },
  "strict_rules": [
    "Use exactly '✅' for correct and '❌' for incorrect.",
    "Do NOT repeat the questions.",
    "Do NOT add explanations beyond what is specified.",
    "Accuracy must be a whole-number percentage rounded to the nearest integer."
  ],
  "output_examples": [
    "1. ✅  (TF)",
    "2. ❌  (FIB) – expected: probe microphone, got: real-ear microphone",
    "3. ✅  (FIB)",
    "Score: 2/3 (Accuracy = 67%)"
  ]
}
```

## N.1.7 Evaluated-model answerers for the objective and research-article tiers

**Two strict-output answerer prompts were used, one per non-case-study tier; both are reproduced verbatim. (a) Educational objective tier (MCQ/TF/FIB) - answerer (`prompt_1_answerer_objective.json`):**

```
You are an expert audiologist.

Task
-----
You will receive a series of JSON objects, one per line, each containing:
  • "type": one of "TF" (true/false), "FIB" (fill-in-the-blank), or "MCQ" (multiple-choice)
  • "question": the text the model must answer.
    For "MCQ", the question text includes the answer options labeled A, B, C, and D.

Output format
-------------
Return **one line per input, in the same order**, following these rules:

1. Begin each line with the 1-based index of the question, followed by a period and a single space.
   Example: `1. `
2. For "TF" questions, write only `True` or `False`.
3. For "FIB" questions, write only the missing word or phrase(s), exactly as they should appear in the blank.
   • If the prompt implies multiple blanks, separate the answers with a comma and a single space (e.g., `auditory, speechreading training`).
4. For "MCQ" questions, write only the single letter (`A`, `B`, `C`, or `D`) corresponding to the correct option.
5. Do **not** repeat, quote, or rephrase the question or provide any explanation.
6. Do **not** output anything before the first answer line or after the last answer line.
```

**(b) Research-article-derived tier (MCQ/TF/SA) - answerer (`prompt_2_answerer_research.json`):**

```
You are an expert audiologist.
Task
-----
You will receive a series of JSON objects, one per line, each containing:
  • "type": one of "TF" (true/false), "MCQ" (multiple choice), or "SA" (short answer)
  • "question": the text the model must answer
Output format
-------------
Return one line per input, in the same order, following these rules:
1. Begin each line with the 1-based index of the question, followed by the item ID and a single space.
2. For "TF" questions, write only "True" or "False" (capitalized exactly).
3. For "MCQ" questions, write only the letter of the correct option (e.g., "A"), capitalized exactly.
   • If the question explicitly allows multiple correct options, list the letters separated by a comma and a single space (e.g., "A, C").
4. For "SA" questions, write a direct, concise answer (≤15 words) that stands on its own.
   • No reasoning, restatements, citations, or qualifiers.
   • If multiple items are explicitly requested, separate them with a comma and a single space.
5. Do not repeat, quote, or rephrase the question; do not provide any explanation.
6. Do not output anything before the first answer line or after the last answer line.
```

## N.1.8 Case-Study Adjudicator Prompt (Claude Opus 4.5, temperature 0)

The following prompt template was applied programmatically to all 804 case-study evaluations (67 cases x 4 evaluated models x 3 question types) using the Anthropic Messages API with model identifier claude-opus-4-5-20251101 and temperature 0. The adjudicator received as input, for each evaluation, a textual reference answer (premise), the evaluated model's response (hypothesis), and a question-type identifier (Q1, Q2, or Q3). The adjudicator did not receive the original diagnostic images, consistent with the reference-answer-based adjudication design described in Methods - Image Handling and Multimodal Adjudication. The adjudicator returned a single JSON object per evaluation with subdimension scores (1-5 scale), critical-error labels, and a free-text justification; the 0-100 weighted score, A-F letter grade, and grade-cap application were computed deterministically from the adjudicator's JSON output using the rubric weights documented below.

### System message

*You are an expert audiologist and clinical documentation specialist serving as an evaluation judge. Your task is to evaluate AI-generated audiology reports by comparing them against ground truth clinical data.*

*You must provide rigorous, objective evaluations based on clinical accuracy, completeness, and safety. Be strict but fair in your assessments.*

### User message template

(Substituted variables shown in {{...}}.)

*## Evaluation Task*

*Evaluate the following AI-generated audiology response (Hypothesis) against the ground truth clinical data (Premise).*

*### Question Type: {{question_type}}*

*{{question_description}}*

*### Case ID: {{case_id}}*

*### Model Being Evaluated: {{model_name}}*

*---*

*### GROUND TRUTH (Premise):*

*{{premise}}*

*---*

*### AI RESPONSE (Hypothesis):*

*{{hypothesis}}*

*---*

*## Evaluation Criteria for {{question_type}}:*

*{{evaluation_criteria}}*

*## Instructions:*

*1. Carefully compare the AI response to the ground truth.*

*2. Evaluate each dimension on a scale of 1-5: 5 = Excellent (accurate, complete, clinically appropriate); 4 = Good (minor omissions or variations, clinically acceptable); 3 = Acceptable (some errors or omissions but generally correct); 2 = Poor (significant errors or missing critical information); 1 = Unacceptable (major errors, contradictions, or dangerous content).*

*3. Identify any CRITICAL ERRORS from the six pre-specified categories: Type Misclassification (SNHL vs CHL vs Mixed); Severity Misclassification (degree off by 2+ categories); Major Hallucination (fabricated test results or data); Laterality Confusion (left/right ear errors); Missed Red Flags (failure to identify urgent conditions); Dangerous Recommendation (clinically harmful advice).*

*4. Provide a brief justification for your scores.*

*## Required Output Format (JSON):*

*{ "case_id": {{case_id}}, "model": "{{model_name}}", "question_type": "{{question_type}}", "dimension_scores": { {{dimension_scores_template}} }, "critical_errors": ["list of critical errors if any, empty array if none"], "justification": "Brief explanation of the evaluation", "overall_assessment": "One of: Excellent, Good, Acceptable, Poor, Unacceptable" }*

*Provide ONLY the JSON output, no additional text.*

### Substituted criteria by question type

Q1 - Audiometric Results:

- Numerical Accuracy (40%): Precision of threshold values, SRT, WRS percentages. Are the dB values within plus or minus 10 dB of ground truth? Are speech scores accurately reported?
- Test Coverage (30%): Completeness of test battery reporting (pure tone audiometry, speech testing, tympanometry, reflexes, OAE, ABR as applicable).
- Description Accuracy (20%): Correct classification of hearing loss type (sensorineural / conductive / mixed), degree (normal / mild / moderate / moderately severe / severe / profound), and configuration (flat / sloping / rising / notched).
- Hallucination-Free (10%): Absence of fabricated data - no invented test results or values not in ground truth.

Q2 - Impression / Diagnosis:

- Diagnostic Accuracy (50%): Correctness of primary diagnosis. Does it identify the correct pathology / condition? Are differential diagnoses appropriate?
- Reasoning Logic (30%): Quality of clinical reasoning. Are conclusions supported by the audiometric data? Is the reasoning clinically sound?
- Evidence Support (20%): Use of test data to support diagnosis. Are specific test results cited appropriately? Is the evidence-to-conclusion chain clear?

Q3 - Recommendations:

- Relevance (40%): Appropriateness of recommendations to diagnosis. Do recommendations address the identified condition? Are they clinically indicated?
- Clinical Feasibility (30%): Practicality and safety. Are recommendations achievable? Are there any potentially harmful suggestions?
- Completeness (30%): Coverage of key recommendation areas (amplification, monitoring, referrals, counseling) as appropriate.

### Dimension-score JSON templates

| **Q-type** | **Subdimension keys (output order in JSON)** |
| --- | --- |
| Q1 | numerical_accuracy, test_coverage, description_accuracy, hallucination_free |
| Q2 | diagnostic_accuracy, reasoning_logic, evidence_support |
| Q3 | relevance, clinical_feasibility, completeness |

### Post-adjudication score computation (deterministic)

The adjudicator's JSON output was processed by a deterministic post-processing script to derive the manuscript-reported scores:

1. Weighted score (1-5 scale) = sum over subdimensions of (subdimension score x subdimension weight). Weights are listed in main-text Table 4 and reproduced verbatim above.

2. Final score (0-100 scale) = weighted score x 20.

3. Grade-cap application: if critical_errors is non-empty, the final score is capped per main-text Table 3: five categories (Type Misclassification, Severity Misclassification, Fabricated or Erroneous Test Result, Laterality Confusion, Missed Red Flag) cap at 49 (grade D); one category (Dangerous Recommendation) caps at 24 (grade F).

4. Letter grade assigned per main-text Table 2 (A: 90-100; B: 75-89; C: 50-74; D: 25-49; F: 0-24).

### Note on rubric-label naming

In the verbatim adjudicator prompt above, the third critical-error category is labelled "Major Hallucination" - the operational identifier used during data collection (November 2025). The released main-text rubric (Table 3) and the public dataset use the canonical clinical label "Fabricated or Erroneous Test Result" for the same category; the definition is unchanged. The rename was applied during dataset normalization for release.

### Evaluation execution

The case-study evaluations were performed in two batches using the same prompt and adjudicator: a 50-case batch in November 2025, and a 17-case batch shortly thereafter. The two batches together cover the full 67-case dataset (n = 804 evaluations). Per-evaluation outputs for both batches are released at the project repository (https://github.com/pc9413/AudiologyQA).

# N.2 Cases Adjudicator Output Schema

For each of the 804 case-study evaluations (67 cases x 4 models x 3 question types), the released per-evaluation outputs (`merged_804_evals.csv`) contain both raw adjudicator fields and derived post-processing fields. Raw adjudicator fields, returned directly by Claude Opus 4.5, are: `dimension_scores`, `critical_errors`, `justification`, and `overall_assessment`. Derived fields, computed deterministically from the raw output using the rubric weights and capping rule documented in Section N.1.8, are: `weighted_score`, `final_score`, `grade`, `score_capped`, `has_ce`, and `passed`. All fields below are released:

- `case_id`: integer 1-67

- `model`: one of {gemini-2.5-pro, grok-4, openai-o3, claude-sonnet-4-thinking}

- `question_type`: one of {Q1, Q2, Q3}

- `dimension_scores`: JSON object of per-subdimension scores on 1-5 integer scale (sub-dimension names by question type listed below)

- `weighted_score`: weighted aggregation of dimension scores per the rubric weights in Table 4 (1-5)

- `final_score`: weighted_score x 20 (0-100)

- `grade`: one of {A, B, C, D, F} per the A–F anchor table in main-text Table 2

- `critical_errors`: list of zero or more pre-specified critical-error type strings drawn from the six categories defined in main-text Table 3 (Type Misclassification, Severity Misclassification, Fabricated or Erroneous Test Result, Laterality Confusion, Missed Red Flag, Dangerous Recommendation)

- `score_capped`: boolean indicating whether the score-capping rule was applied (true when a critical error of category capped-at-D or capped-at-F was triggered)

- `has_ce`: boolean derived from `len(critical_errors) > 0`

- `passed`: boolean derived from `grade in {A, B, C}`

## N.2.1 Sub-dimensions by question type

Q1 (Audiometric Numerical Interpretation):

- numerical_accuracy (1-5)
- test_coverage (1-5)
- description_accuracy (1-5)
- hallucination_free (1-5)

Q2 (Diagnostic Reasoning):

- diagnostic_accuracy (1-5)
- reasoning_logic (1-5)
- evidence_support (1-5)

Q3 (Clinical Recommendations):

- relevance (1-5)
- clinical_feasibility (1-5)
- completeness (1-5)

# N.3 Audit Agreement - Full Per-Stratum Tables

## Table S1. Inter-reviewer and adjudicator-vs-consensus agreement, per-stratum

| **Comparison** | **Stratum** | **n** | **Grade QW-κ** | **95% CI** |
| --- | --- | --- | --- | --- |
| Inter-reviewer (R88 vs R33) | Pre-consensus, all | 50 | 0.85 | 0.71-0.94 |
| Inter-reviewer (R88 vs R33) | Calibration segment (1-8) | 8 | 0.84 | — |
| Inter-reviewer (R88 vs R33) | Main segment (9-50) | 42 | 0.86 | — |
| Adjudicator vs consensus | All 50 items | 50 | 0.31 | 0.03-0.55 |
| Adjudicator vs consensus | Q1 stratum | 17 | -0.14 | — |
| Adjudicator vs consensus | Q2 stratum | 16 | 0.57 | — |
| Adjudicator vs consensus | Q3 stratum | 17 | 0.52 | — |
| Adjudicator vs consensus | Reviewer-agreement subset | 41 | 0.43 | 0.16-0.66 |
| Adjudicator vs consensus | Reviewer-agreement Q1 subset | 11 | -0.11 | — |
| Adjudicator vs consensus | Reviewer-agreement Q2 subset | 13 | 0.62 | — |
| Adjudicator vs consensus | Reviewer-agreement Q3 subset | 17 | 0.55 | — |
| Inter-reviewer (any-CE flag) | Pre-consensus | 50 | 0.59 (unweighted κ) | 0.34-0.83 |
| Adjudicator vs consensus (any-CE) | All 50 items | 50 | 0.10 (unweighted κ) | -0.19 to +0.41 |

## Table S2. ICC(2,1) absolute agreement on grade-midpoint mapped scores

Grade-midpoint mapping: A = 97.3, B = 82.0, C = 60.6, D = 45.0.

| **Comparison** | **n** | **ICC(2,1)** | **95% CI** |
| --- | --- | --- | --- |
| R88 vs R33 | 50 | 0.81 | 0.69-0.89 |
| Adjudicator vs human consensus | 50 | 0.31 | 0.05-0.53 |

## Table S3. Six alternative consensus rules - judge-vs-consensus grade QW-κ

Sensitivity check confirming that the adjudicator-vs-consensus agreement result is robust to consensus-formation rule choice.

| **Consensus rule** | **Grade QW-κ** | **95% CI** |
| --- | --- | --- |
| R88 only | 0.34 | 0.05-0.58 |
| R33 only | 0.31 | 0.03-0.54 |
| Pre-adjudication strict (both must agree) | 0.31 | 0.04-0.55 |
| Pre-adjudication lenient (either can flag) | 0.32 | 0.05-0.56 |
| Pre-adjudication mean (grade midpoint average) | 0.33 | 0.06-0.57 |
| Final consensus (study team adjudication) | 0.31 | 0.03-0.55 |

# N.4 Calibration Map and Calibrated Q-Type Means

## Table S4. 12-cell calibration map

Raw cell delta is defined as mean LLM-judge final_score minus mean human-consensus mapped score within the audited cell, computed on the 50-item audit subsample. For sparse cells with n < 3 audited items, the applied calibration delta uses the question-type-level mean item delta (fallback) rather than the within-cell raw delta. Q-type-level mean item deltas (used as fallback): Q1 = +6.70, Q2 = −6.91, Q3 = −7.08. Human-calibrated score was computed as human_calibrated_score = clip(original_LLM_score − applied_delta, 0, 100).

| **Q-type x judge-grade cell** | **n audited** | **LLM-judge mean** | **Human consensus mean** | **Applied calibration delta** |
| --- | --- | --- | --- | --- |
| Q1 - A | 4 | 97.50 | 63.50 | +34.00 |
| Q1 - B | 4 | 83.13 | 67.40 | +15.73 |
| Q1 - C | 4 | 65.00 | 54.25 | +10.75 |
| Q1 - D | 5 | 49.00 | 74.60 | −25.60 |
| Q2 - A | 1 (fallback) | 95.00 | 97.30 | −6.91 (Q2 fallback) |
| Q2 - B | 7 | 81.43 | 81.09 | +0.34 |
| Q2 - C | 4 | 55.63 | 67.40 | −11.78 |
| Q2 - D | 4 | 42.25 | 58.15 | −15.90 |
| Q3 - A | 5 | 97.00 | 94.24 | +2.76 |
| Q3 - B | 4 | 80.63 | 80.48 | +0.15 |
| Q3 - C | 4 | 63.13 | 62.05 | +1.08 |
| Q3 - D | 4 | 41.88 | 76.65 | −34.78 |

Cell-level values are derived from the 50-item audit subsample (n = 17 Q1, 16 Q2, 17 Q3). The full reproducible derivation, including item-level cell membership and the application of cell deltas to all 804 evaluations, is provided by the analysis script `audit_calibration_analysis.py` (released alongside `audit_agreement_analysis.py`).

## Table S5. Q-type means under original LLM-judge and human-calibrated mappings

| **Q-type** | **Original LLM-judge mean** | **Human-calibrated mean (sensitivity)** |
| --- | --- | --- |
| Q1 (n=268) | 67.89 | 65.19 |
| Q2 (n=268) | 67.79 | 74.05 |
| Q3 (n=268) | 89.74 | 88.38 |

Q3 > {Q1, Q2} ranking is preserved under human-calibrated mapping. The most material shift is upward movement of Q2 relative to Q1 under human scoring. Under all three sparse-cell handling rules the Q3 > {Q1, Q2} ranking is preserved: the question-type fallback used above (Q1/Q2/Q3 = 65.19/74.05/88.38), dropping the sparse cell (65.19/74.01/88.38), and a question-type-mean-only map (61.19/74.68/93.33). The single sub-threshold cell (Q2 grade-A, n = 1 audited) affects only 2 of the 804 evaluations.

# N.5 Critical-Error Reclassification Audit Trail

Two items in the 50-item audit subsample (S22, S32) were initially marked 'no critical error' by both independent reviewers and were reclassified as critical-error cases under consensus adjudication.

## S22

Original adjudicator label: critical error - Fabricated or Erroneous Test Result.

Initial reviewer labels: R88 = no critical error; R33 = no critical error.

Consensus rationale: ABR air-conduction thresholds and air-bone gaps in the model response were substantially understated relative to the textual reference (numerical fabrication), simultaneously triggering Fabricated or Erroneous Test Result and ≥ 2-category Severity Misclassification per Table 3 definitions. Reclassified as critical-error: yes.

## S32

Original adjudicator label: critical error - Type Misclassification.

Initial reviewer labels: R88 = no critical error; R33 = no critical error.

Consensus rationale: traumatic ossicular-disarticulation conductive loss labeled as sensorineural in the model response. Reclassified as critical-error: yes.

# N.6 Sub-Dimension Means by Question Type and Model

Computed from the 804 per-evaluation outputs. All values on 1-5 scale per sub-dimension.

## Q1 sub-dimensions

| **Model** | **numerical_accuracy** | **test_coverage** | **description_accuracy** | **hallucination_free** |
| --- | --- | --- | --- | --- |
| Gemini 2.5 Pro | 3.97 | 4.33 | 4.13 | 3.97 |
| Grok 4 | 3.42 | 4.24 | 4.03 | 3.91 |
| OpenAI O3 | 3.88 | 4.54 | 3.82 | 3.94 |
| Claude Sonnet 4 Thinking | 3.58 | 4.51 | 3.99 | 3.94 |

Q1 numerical_accuracy is uniformly the lowest sub-dimension across all four models (range 3.42-3.97); test_coverage is uniformly the highest (range 4.24-4.54). This pattern explains the Q1 critical-error rate concentration on Fabricated or Erroneous Test Result and Severity Misclassification: models can describe which audiometric tests were performed but mis-state the numerical values.

## Q2 sub-dimensions

| **Model** | **diagnostic_accuracy** | **reasoning_logic** | **evidence_support** |
| --- | --- | --- | --- |
| Gemini 2.5 Pro | 4.01 | 4.61 | 3.04 |
| Grok 4 | 3.97 | 3.43 | 3.03 |
| OpenAI O3 | 3.79 | 3.69 | 3.03 |
| Claude Sonnet 4 Thinking | 3.78 | 3.78 | 3.01 |

Evidence_support is uniformly the lowest Q2 sub-dimension (3.01-3.04) across models. Gemini 2.5 Pro's Q2 advantage comes specifically from reasoning_logic (4.61 vs 3.43-3.78 for others).

## Q3 sub-dimensions

| **Model** | **relevance** | **clinical_feasibility** | **completeness** |
| --- | --- | --- | --- |
| Gemini 2.5 Pro | 4.46 | 4.69 | 4.46 |
| Grok 4 | 4.61 | 4.66 | 4.61 |
| OpenAI O3 | 4.40 | 4.84 | 4.40 |
| Claude Sonnet 4 Thinking | 4.70 | 4.60 | 4.70 |

All Q3 sub-dimensions ≥ 4.40 across all models, consistent with the 89.74 overall Q3 mean and 98.1% pass rate.

# N.7 Case-Level Sensitivity Analyses

## N.7.1 Mixed-effects model

Specification: final_score ~ question_type + (1 | case_id), REML.

| **Parameter** | **Estimate** | **SE** | **p-value** |
| --- | --- | --- | --- |
| Q1 intercept (Q1 mean) | 67.89 | 1.20 | < .001 |
| Q2 - Q1 coefficient | -0.10 | 1.26 | 0.94 |
| Q3 - Q1 coefficient | 21.85 | 1.26 | < .001 |

Variance components: Var(case random intercept) = 43.83; Var(residual) = 213.35; Var(fixed effects) = 106.60.

Marginal R² (fixed only) = 0.293; Conditional R² (fixed + random) = 0.414; ICC = 0.170.

Interpretation: the case-level random-intercept variance accounts for approximately 17% of the within-question-type variance, consistent with moderate but limited case-level clustering. The Q3 vs Q1 fixed-effect coefficient remains highly significant under random-effects accounting.

## N.7.2 Case-level non-parametric bootstrap (B = 1,000)

Resample 67 cases with replacement; recompute Q-type means each iteration. Seed = 20260428.

| **Metric** | **Median** | **95% bootstrap CI** |
| --- | --- | --- |
| Q1 mean | 67.96 | [64.74, 70.94] |
| Q2 mean | 67.78 | [65.04, 70.44] |
| Q3 mean | 89.73 | [87.26, 92.07] |
| Q3 - Q1 difference | 21.82 | [18.38, 25.53] |
| Q3 - Q2 difference | 21.98 | [18.93, 24.84] |
| Q1 - Q2 difference | -0.09 | [-3.68, +3.71] |

Directional consistency: Q3 > Q1 in 1,000 / 1,000 bootstraps (100%); Q3 > Q2 in 1,000 / 1,000 bootstraps (100%).

## N.7.3 Leave-one-case-out (LOOCV)

Excluded each of 67 cases in turn (67 LOOCV folds).

| **Metric** | **Min across folds** | **Max across folds** | **Spread** |
| --- | --- | --- | --- |
| Q1 mean | 67.44 | 68.19 | 0.75 |
| Q2 mean | 67.51 | 68.13 | 0.62 |
| Q3 mean | 89.58 | 90.20 | 0.62 |

Directional consistency: Q3 > Q1 in 67/67 folds (100%); Q3 > Q2 in 67/67 folds (100%). No single case drives the Q-type ranking.

# N.8 Shared-Failure Tabulation

Per-case fail-count distribution across 4 evaluated models, stratified by question type. Fail = grade D (which includes any score-capped critical-error case).

## Table S6. Number of cases at which N of 4 models fail

| **Q-type** | **0 fail** | **1 fails** | **2 fail** | **3 fail** | **4 fail** | **Total** |
| --- | --- | --- | --- | --- | --- | --- |
| Q1 | 25 | 14 | 12 | 5 | 11 | 67 |
| Q2 | 59 | 3 | 1 | 3 | 1 | 67 |
| Q3 | 63 | 3 | 1 | 0 | 0 | 67 |

## Independence test

Under independence of model failures within a case (H₀), expected count of all-four-fail cases by question type:

| **Q-type** | **Marginal fail rate p** | **Expected all-fail (= 67p^4)** | **Observed all-fail** |
| --- | --- | --- | --- |
| Q1 | 0.362 | 1.15 | 11 |
| Q2 | 0.067 | 0.00 | 1 |
| Q3 | 0.019 | 0.00 | 0 |

Q1 observed all-fail count (11) is approximately 10x the count expected under independence (1.15) - strong evidence of shared-failure structure on audiometric numerical interpretation.

## All-fail Q1 case_ids

5, 13, 14, 23, 25, 30, 31, 37, 39, 53, 63

# N.9 Rubric Reference Table

Reproduces and expands main-text Table 2 (A–F anchor descriptions) and Table 3 (six pre-specified critical-error types and grade caps). The sub-dimension definitions used by the case-study adjudicator are documented below.

## Q1 (Audiometric Numerical Interpretation) sub-dimension definitions

- numerical_accuracy: extent to which audiometric values reported by the model (thresholds, SRT, WRS, tympanometry, ABR/OAE outputs) match the textual reference values.
- test_coverage: whether the model identifies and reports all clinically relevant audiometric tests present in the case.
- description_accuracy: accuracy of test-interpretation descriptions (e.g., type, severity, configuration of hearing loss; tympanogram type).
- hallucination_free: absence of fabricated tests, fabricated values, or values inconsistent with the textual reference.

## Q2 (Diagnostic Reasoning) sub-dimension definitions

- diagnostic_accuracy: correctness of the differential diagnosis or impression relative to the case-history reference.
- reasoning_logic: internal coherence of the diagnostic argument.
- evidence_support: extent to which the diagnostic conclusion is supported by case-specific findings cited in the response.

## Q3 (Clinical Recommendations) sub-dimension definitions

- relevance: alignment of recommendations with the diagnosed problem and case context.
- clinical_feasibility: practical implementability and standard-of-care alignment.
- completeness: coverage of expected recommendation domains (amplification, medical referral, follow-up, counseling, assistive technology).

## Critical-error type definitions (replicates main-text Table 3)

- Type Misclassification (capped at D): hearing loss type assigned incorrectly (e.g., conductive labeled as sensorineural).
- Severity Misclassification (capped at D): hearing loss degree differs from reference by ≥ 2 categories on the standard severity scale.
- Fabricated or Erroneous Test Result (capped at D): test results reported in the model response that contradict or are not present in the reference.
- Laterality Confusion (capped at D): bilateral vs unilateral mismatch, or right/left side swap.
- Missed Red Flag (capped at D): failure to identify or address an urgent finding warranting referral or precaution (e.g., asymmetric SNHL, sudden SNHL within corticosteroid window, ototoxicity monitoring requirement, post-traumatic conductive loss).
- Dangerous Recommendation (capped at F): active recommendation that, if followed, would directly cause patient harm or contraindicate appropriate care.

The asymmetric grade-cap (D for Missed Red Flag vs F for Dangerous Recommendation) reflects the conceptual division between commission and omission errors: a Dangerous Recommendation introduces a positive harmful action; a Missed Red Flag fails to recommend an escalation that would normally be caught at the next step in clinical workflow.

# N.10 Effect-Size Concordance: Cohen's d vs Rank-Biserial r

Concordance between the parametric (Cohen's d) and non-parametric (rank-biserial r) effect-size metrics across all reported pairwise contrasts. Rank-biserial r is reported as the primary effect size in the revised manuscript (consistent with the Mann-Whitney U testing framework); Cohen's d is retained as a supplementary descriptive metric for comparability with the broader LLM evaluation literature.

## Table S7. Effect-size concordance

| **Contrast** | **n1** | **n2** | **U** | **Rank-biserial r** | **Cohen's d** | **Interpretation** |
| --- | --- | --- | --- | --- | --- | --- |
| Q1 vs Q3 (case-study) | 268 | 268 | 11,540 | 0.679 (very large) | 1.34 (very large) | Q3 > Q1 |
| Q2 vs Q3 (case-study) | 268 | 268 | 9,692 | 0.730 (very large) | 1.50 (very large) | Q3 > Q2 |
| Q1 vs Q2 (case-study) | 268 | 268 | 33,573 | 0.065 (negligible) | 0.006 (negligible) | Q1 ≈ Q2 |
| Gemini vs Grok (Q2) | 67 | 67 | 3,082 | 0.373 (medium) | 0.635 (medium-large) | Gemini > Grok |
| Gemini vs O3 (Q2) | 67 | 67 | 3,081 | 0.373 (medium) | 0.664 (medium-large) | Gemini > O3 |
| Gemini vs Sonnet (Q2) | 67 | 67 | 3,068 | 0.367 (medium) | 0.632 (medium-large) | Gemini > Sonnet |

KW omnibus η²_H values (matches main-text reported η²): question type η²_H = (268.44 - 2)/(804 - 3) = 0.333; model omnibus η²_H = (3.66 - 3)/(804 - 4) = 0.001; Q2-stratified model effect η²_H = (21.16 - 3)/(268 - 4) = 0.069.

Across all six contrasts, the rank-biserial r and Cohen's d agree on directional sign and qualitative effect-size category (negligible / medium / very large).

# N.11 Audit Dataset, Analysis Script, and Provenance

The frozen audit dataset (v1.0) is publicly available at the project repository (https://github.com/pc9413/AudiologyQA). The repository contains:

- `merged_804_evals.csv`: per-evaluation outputs for all 804 case-study evaluations, comprising raw Claude Opus 4.5 adjudicator fields (per-subdimension scores, critical-error type list, justification, overall assessment) and derived post-processing fields (weighted score, final score, grade, score-capping flag, has-CE, passed booleans); see Section N.2 for the full schema.
- `audit_50_dataset_v1.0.csv`: the 50-item human-audit subsample frozen for reliability analysis.
- `audit_R88_labels.csv` and `audit_R33_labels.csv`: independent audiologist reviewer labels for the 50-item subsample.
- `audit_consensus_labels.csv`: consensus-resolved labels for the 22 items requiring adjudication, plus the 28 reviewer-agreed items.
- `audit_rationales.csv`: AI-drafted rationales with study-lead attestations (5 modify, 3 spot-check, 17 first-pass-screened).
- `audit_calibration_map.csv`: 12-cell (Q-type x adjudicator-grade) calibration map.
- `audit_calibrated_804_means.csv`: Q-type x Model means under the human-calibrated mapping.
- `audit_agreement_analysis.py`: Python analysis script reproducing all inter-reviewer kappa values (R88 vs R33 grade QW κ = 0.85; any-CE κ = 0.59), adjudicator-vs-consensus kappa (grade QW κ = 0.31; Q1 stratum QW κ = -0.14), ICC values, bootstrap CIs, and calibrated means from the above CSVs.

Analysis-script deterministic seeds: kappa bootstrap = 20260427; case-level bootstrap = 20260428.

The revised submission additionally releases the raw artefacts for the expanded human-audit components: the reviewer labels for the expanded, image-grounded Q1 audit (Section N.15); the within-task human baseline answers and adjudicator-pipeline scores (Section N.16); the 210-item reference-answer fidelity labels and the 1,460-item reference-grounding scan (Section N.17); the multi-pass NLI scores (mednli_scores_17.csv; Section N.14); and a consolidated reproducible analysis script (E_analysis_R2_canonical.py), together with audit_calibration_analysis.py, that regenerates the reliability, baseline, fidelity, and calibration statistics reported above.

# N.12 Representative Item Examples

This section provides one representative item from each of the six format x source-pool combinations released as part of the AUDIOLOGYBENCH dataset, with reference answers and adjudication paths annotated. Educational-pool items are released in `AudiologyQA.jsonl`; research-article-derived items are released in the `Audiology/Hearing Sciences` domain subset of `research_article_QA_labeled.jsonl`. All items below are reproduced verbatim from the released dataset.

## N.12.1 Educational-pool MCQ (item ID `MC94`; Clinical Decision-Making, hard)

Stem: "In the context of electrophysiological assessments, what does the presence of a cochlear microphonic in the absence of wave I in an ABR suggest?" Options: A) Normal auditory nerve function; B) Conductive hearing loss; C) Presence of auditory neuropathy spectrum disorder; D) Middle ear pathology.

Reference answer: C. Adjudication: deterministic exact-match against the reference option letter; no LLM-judge call.

## N.12.2 Educational-pool TF (item ID `TF823`; Anatomy & Physiology, medium)

Stem: "Each subdivision of the Cochlear Nucleus (CN) is highly tonotopic, with low frequencies represented in the dorsal portion and high frequencies in the ventral portion."

Reference answer: False. Adjudication: deterministic case-insensitive exact-match against the True/False reference; no LLM-judge call.

## N.12.3 Educational-pool FIB (item ID `FIB474`; Instrumentation, medium)

Stem: "__________ is used in implantable hearing technologies to deliver signals via radiofrequency transmission and to transduce signals between states like magnetic and electrical current."

Reference answer: "Electromagnetic induction". Adjudication: Gemini 2.5 Pro LLM-judge at temperature 0 under the binary rubric in `prompt_2_grader.json`.

Acceptable variant examples illustrating each rubric rule (per `prompt_2_grader.json`): "electromagnetic induction" (rule 1: normalized exact match after lowercasing); "Electromagnetic induction (EMI)" (rule 3: parenthetical acronym difference); "Electromagnetic induction technology" (rule 4: generic filler-noun difference); "EM induction" (rule 5: expert-judgment true synonym in standard audiology usage).

## N.12.4 Research-article-derived MCQ (item ID `1614`; Hearing Technology, Intermediate)

Source paper: Rodman C, Moberly AC, Janse E, Baskent D, Tamati TN. The impact of speaking style on speech recognition in quiet and multi-talker babble in adult cochlear implant users. J Acoust Soc Am. 2020;147(1):101-107.

Stem: "For which group of cochlear implant (CI) users does clear speech facilitate recognition in multi-talker babble?" Options: A. All CI users equally; B. Lower-performing CI users; C. Higher-performing CI users; D. Only CI users with residual hearing.

Reference answer: C. Adjudication: deterministic exact-match against the reference option letter; no LLM-judge call.

## N.12.5 Research-article-derived TF (item ID `4015`; Hearing Technology, Advanced)

Source paper: Brodie KD, Florentine MM, Sabbagh S, Chan DK. Impact of Genetic Testing on Hearing Interventions. Laryngoscope. 2023;133(8):1982-1986.

Stem: "Children with Sensorineural Hearing Loss (SNHL) who receive a specific genetic diagnosis are significantly more likely to subsequently utilize hearing aids or undergo cochlear implantation."

Reference answer: True. Adjudication: deterministic case-insensitive exact-match against the True/False reference; no LLM-judge call.

## N.12.6 Research-article-derived SA (item ID `1310`; Clinical Audiology, Advanced)

Source paper: Huang H, Chen YF, Hsu CY, Cheng YF, Yang TH. Evaluating auditory brainstem response to a level-dependent chirp designed based on derived-band latencies. J Acoust Soc Am. 2022;151(4):2688.

Stem: "A level-dependent derived-band (DB)-Chirp was designed and compared to a level-dependent level specific (LS)-Chirp and a click for evoking auditory brainstem responses (ABRs). At what level did the DB-Chirp produce a significantly larger wave V than the LS-Chirp?"

Reference answer: "45 dB normal hearing level (nHL)". Adjudication: Gemini 2.5 Pro LLM-judge at temperature 0 under the binary rubric in `prompt_2_grader.json`.

Acceptable variant examples illustrating rubric rules (per `prompt_2_grader.json`): "45 dB nHL" (rule 3: parenthetical acronym/abbreviation difference); "45 dB normal hearing level" (rule 1: normalized exact match without the parenthetical acronym); "45dB nHL" (rule 1: normalized exact match permitting whitespace variation).

# N.13 Extended Limitations

This section reports additional methodological limitations referenced in the main-text Limitations subsection. Each sub-point is presented in the same form as if retained in the main text; the move to the appendix reflects length-management rather than reduced importance.

## N.13.1 Reference-Answer Fidelity on the Research-Article-Derived Items

The 3,175 research-article-derived items underwent two-expert review during item generation. To test whether the lower short-answer accuracies reflect genuine model failure rather than reference-answer noise, we subsequently audited a stratified, failure-enriched 210-item short-answer sample for reference-answer fidelity (the reference-answer fidelity audit, Section N.17); this audit found no flawed or indeterminate keys and bounded any answer-key or grader artefact at roughly 2–3 percentage points. It was, however, a 210-item sample reviewed by a single doctoral-level audiologist with an independent LLM screen, not a complete multi-rater human audit of all 3,175 research-derived items or all 1,460 short-answer items; residual undetected reference-answer noise on the unaudited remainder therefore cannot be excluded. We accordingly continue to position the research-derived short-answer accuracies as descriptive rather than inferentially compared, now bounded by this fidelity estimate. A full multi-rater human audit across the complete research-derived item set remains an appropriate next study.

## N.13.2 Closed-Book Evaluation

Models were evaluated without retrieval augmentation, tool use, or chain-of-verification scaffolding. Our findings characterize closed-book base capability and should not be extrapolated to deployment configurations that incorporate retrieval, agentic tool use, or post-hoc verification, which may substantially alter performance. Retrieval-augmented and tool-use configurations characterize a methodologically distinct deployment regime—what retrieval recovers from base failure modes—and are appropriately scoped to dedicated follow-up evaluation.

## N.13.3 Self-Preference on the Secondary Tiers

Gemini 2.5 Pro served as both adjudicator and evaluated model on the secondary objective-item and research-article-derived item tiers. Self-preference cannot be excluded for those secondary endpoints. The case-study tier (primary endpoint) is unaffected because Claude Opus 4.5 served as the cases adjudicator and is not among the four evaluated cases models.

## N.13.4 Statistical Analysis Structure

Statistical analyses treated the 804 evaluations as independent observations. The case-level ICC under the one-way model (0.094) and under the mixed-effects model (0.170), together with the very large effect size disparity between question type (eta-squared_H = 0.333; rank-biserial r >= 0.679 for Q1 vs Q3 and Q2 vs Q3) and model (eta-squared_H = 0.001) support directional robustness, but mixed-effects models would more rigorously account for the nested structure. Relevant case-level sensitivity analyses are reported elsewhere in this Appendix (Section N.7).

## N.13.5 Commercial Deployment Context

Commercial LLM applications often incorporate additional safety measures, fine-tuning, retrieval augmentation, and content filters that are not present in base-model API access. Our results characterize base-model capability and may understate performance achievable in product-grade deployments.

## N.13.6 Source-Pool Difficulty Asymmetry and Contamination

Near-ceiling accuracy on web-style MCQ items (>95%) raises the possibility of training-data contamination on the secondary tier and is consistent with the typically lower discriminative difficulty of recall-oriented questions found on open educational websites and Q&A repositories. The lower textbook-sourced MCQ accuracies (35-72%) are interpreted as reflecting item difficulty and curation depth rather than source recency: chapter-end review questions are predominantly written at integration- and application-cognitive levels rather than recall, the textbook source pool was drawn from titles all published from 2015 onward, and the core audiology content tested in these items (e.g., audiometric threshold interpretation, hearing-loss type and severity classification, basic differential diagnosis) is stable across this publication window. We therefore do not interpret source recency as the dominant driver of the online-vs-textbook performance spread reported in Figure 2.

## N.13.7 Cultural and Linguistic Bias - Three-Point Breakdown

Referenced from main-text Limitations §Sample-frame cultural and linguistic bias. The rubric inherits clinical assumptions of the US/Australian English-language source materials in three identifiable respects. First, the severity-classification anchors that underpin the Severity Misclassification critical-error category reflect the multi-band severity scheme used in the source materials; clinical systems applying alternative severity partitions may yield correct interpretations that the rubric labels as a misclassification, and conversely. Second, the Missed Red Flag category is operationalized against US/Australian referral pathways (e.g., audiologist-initiated referral to otolaryngology for asymmetric sensorineural loss, sudden sensorineural loss within the corticosteroid treatment window, and ototoxicity monitoring), which encode a service model in which audiologists hold front-line diagnostic responsibility. Third, the Q3 recommendation space (amplification, medical referral, follow-up, counseling, assistive technology) reflects scope-of-practice norms of audiologist-led independent practice and may not capture the constrained recommendation space of ENT-led, dispenser-led, or community-health-worker-led service models.

## N.13.8 Inter-Rater Reliability Records on the Objective and Research-Article-Derived Items

Referenced from main-text Methods — Scoring and Preprocessing — Objective Items. Two-expert review of the 3,139 educational and 3,175 research-article-derived items was conducted concurrently rather than as an independent blinded second pass; only post-consensus revised or removed items were preserved, and formal pre-consensus inter-rater agreement records (e.g., Cohen’s κ between independent reviewers, or between human reviewers and the Gemini 2.5 Pro automated adjudicator on the FIB and SA grading paths) were not retained. This precludes a formal κ-based estimate of reviewer noise on the secondary tiers. The case-study primary endpoint, on which the inferential framework is built, has full pre-consensus and post-consensus reliability documented in Methods — Human Audit of Automated Adjudication and Results — Audit Agreement (grade quadratic-weighted κ = 0.85 between the two human reviewers; adjudicator-vs-consensus κ = 0.31). A blinded second-pass reliability audit of the objective and research-article-derived item adjudication is identified as an appropriate methodological refinement for follow-up work.

# N.14 Multi-Pass Stochastic Stability of the Task-Type Dissociation

To verify that the Q1 audiometric-interpretation failure is a stable property of model generation rather than an artefact of single-pass sampling, each of the four case-study models was re-run ten times per case–question on a 17-case stability subsample (17 cases × 4 models × 3 question types × 10 passes = 2,040 responses), under the same default API settings used for the primary evaluation. Every response was scored against the frozen per-(case, question) reference answer by a deterministic medical natural-language-inference (NLI) classifier that labels each response as entailment, neutral, or contradiction relative to the reference. Because the classifier is deterministic, all pass-to-pass variation in its labels reflects stochastic variation in model generation rather than scorer noise.

The contradiction-versus-reference rate is used here as a model-blind, judge-independent index of cross-pass stability. It is computed independently of the rubric-based automated adjudicator (Claude Opus 4.5), and the NLI scorer never receives a model identifier; the rate is therefore not a re-estimate of the 35.4% rubric critical-error rate but a stability check on the task-type dissociation.

**Table N.14.1 Contradiction-versus-reference rate by question type (10 passes pooled; 17-case subsample).**

| **Question type** | **Contradiction-vs-reference rate** | **n (responses)** |
| --- | --- | --- |
| Q1 (Results) | 29.7% | 680 |
| Q2 (Impression/Diagnosis) | 31.0% | 680 |
| Q3 (Recommendations) | 2.8% | 680 |

**Table N.14.2 Cross-pass stability of the Q1 contradiction rate.**

| **Quantity** | **Value** |
| --- | --- |
| Q1 contradiction rate, mean across 10 passes | 29.7% |
| Q1 contradiction rate, SD across passes | 3.1 percentage points |
| Q1 contradiction rate, range across passes | 25.0–35.3% |
| Per-pass Q1 rates (passes 1–10) | 32.4, 26.5, 25.0, 35.3, 29.4, 27.9, 32.4, 30.9, 29.4, 27.9% |
| Q1 model-case cells with the same label in ≥ 8 of 10 passes | 71% (48/68) |
| Q1 model-case cells with the same label in ≥ 9 of 10 passes | 51% (35/68) |
| Q1 model-case cells identical across all 10 passes | 34% (23/68) |

**Table N.14.3 Q1 and Q3 contradiction rates by model (10 passes pooled).**

| **Model** | **Q1 contradiction rate** | **Q3 contradiction rate** |
| --- | --- | --- |
| Gemini 2.5 Pro | 14.1% | 2.4% |
| Grok 4 | 44.1% | 2.4% |
| OpenAI O3 | 29.4% | 2.4% |
| Claude Sonnet 4 Thinking | 31.2% | 4.1% |

Interpretation. The Q1 contradiction rate is stable across the ten passes (mean 29.7%, SD 3.1 percentage points, range 25.0–35.3%), and a majority of Q1 model-case items receive the same label in at least 8 of 10 passes (71%). The Q1 ≫ Q3 task-type dissociation is reproduced under this judge-independent, model-blind scorer within every evaluated model (Q3 contradiction 2.8% overall, ≤ 4.1% for every model), mirroring the rubric-based finding in the main analysis. The 35.4% Q1 critical-error rate reported in the main text therefore reflects a stable failure mode rather than single-pass stochastic variance. All values are reproduced by the released analysis over mednli_scores_17.csv.

*Expanded human-audit additions for the revised manuscript. All values are reproduced by the released analysis script (E_analysis_R2_canonical.py). The audiologist reviewers were study co-authors, blinded to model identity and to the automated adjudicator's labels; for the within-task baseline, reviewers answered each case under closed-book conditions before any model-response grading.*

# **N.15 Expanded Image-Grounded Q1 Audit: reviewer and adjudicator reliability**

Two PhD-level audiologists (study co-authors) independently re-graded all 80 model Q1 (audiometric-interpretation) responses from the audit sample against Tables 2–4, with the diagnostic audiogram images available. The 80 responses were drawn under a stratified scheme enriched for all-four-model-failure cases (44 of 80 items from all-fail cases; 36 from paired control cases). All population-level rates are reweighted to the 268-evaluation Q1 population using per-stratum analysis weights (values 1.00, 5.00, 6.00, 6.25, 7.00). Confidence intervals are nonparametric bootstrap (B = 2000) for kappa and detection metrics; the weighted rate intervals are bootstrap over the reweighted sample.

**Table N.15.1 Reliability of the expanded 80-item Q1 audit.**

| **Comparison** | **Metric** | **Value (95% CI)** |
| --- | --- | --- |
| Inter-reviewer (two audiologists) | Grade quadratic-weighted kappa | 0.78 (0.68–0.85) |
| Inter-reviewer (two audiologists) | Any-critical-error kappa | 0.91 (0.80–1.00) |
| Inter-reviewer (two audiologists) | Exact grade agreement | 65% |
| Inter-reviewer (two audiologists) | Within-one-grade agreement | 99% |
| Adjudicator vs human reviewers | Grade quadratic-weighted kappa | 0.05 (−0.06 to 0.16) |
| Adjudicator vs human reviewers | Critical-error sensitivity | 73% (55–89) |
| Adjudicator vs human reviewers | Critical-error specificity | 37% (25–51) |
| Adjudicator vs human reviewers | Critical-error positive predictive value | 36% (24–49) |
| Adjudicator vs human reviewers | Over-flags / under-detections (counts) | 34 / 7 |

**Table N.15.2 Q1 critical-error rate reweighted to the 268-evaluation population.**

| **Source** | **Weighted Q1 critical-error rate** |
| --- | --- |
| Automated adjudicator (text-only) | 36.2% |
| Audiologist 1 (image-grounded) | 28.3% |
| Audiologist 2 (image-grounded) | 33.6% |
| Range across label-combination rules | 28–34% |

Interpretation. Inter-reviewer reliability is high and stable. The adjudicator's per-item Q1 labels diverge from human judgment (grade quadratic-weighted kappa = 0.05) and the direction of error is over-flagging (high sensitivity, low specificity/PPV), superseding the unstable n = 17 estimate of −0.14 from the original 50-item audit. The earlier overall sensitivity of 25% rested on only 12 critical-error-positive items pooled across question types and is not directly comparable to this Q1-specific estimate. Despite the per-item noise, the adjudicator's reweighted aggregate rate (36.2%) is consistent with the image-grounded human estimate (28–34%), so the headline 35.4% shows no evidence of systematic inflation; because the human reviewers had the diagnostic images and the adjudicator did not, this also indicates that text-only adjudication does not materially distort the aggregate Q1 rate.

# **N.16 Within-Task Human Baseline**

Two PhD-level audiologists (study co-authors) independently answered Q1/Q2/Q3 on 16 case studies under closed-book conditions, before any model-response grading. The 16 cases were deliberately enriched for model failure (11 of 16 are cases on which all four models produced a Q1 critical error). Human answers were scored by the identical automated adjudicator pipeline used for the models, blinded to whether each answer was human- or model-generated. Critical-error-rate confidence intervals are Wilson; combined mean-score confidence intervals are case-level nonparametric bootstrap (B = 5000, seed 94755).

**Table N.16.1 Mean score, pass rate, and critical-error rate by source and question type (16 cases).**

| **Source** | **Q** | **Mean** | **Pass** | **CE (n)** | **CE rate [95% CI]** |
| --- | --- | --- | --- | --- | --- |
| Audiologist 1 | Q1 | 66.8 | 94% | 1/16 | 6.2% [1.1-28.3] |
| Audiologist 1 | Q2 | 62.1 | 69% | 1/16 | 6.2% [1.1-28.3] |
| Audiologist 1 | Q3 | 91.7 | 94% | 1/16 | 6.2% [1.1-28.3] |
| Audiologist 2 | Q1 | 69.8 | 100% | 0/16 | 0.0% [0.0-19.4] |
| Audiologist 2 | Q2 | 75.9 | 94% | 1/16 | 6.2% [1.1-28.3] |
| Audiologist 2 | Q3 | 95.2 | 100% | 0/16 | 0.0% [0.0-19.4] |
| Gemini 2.5 Pro | Q1 | 55.4 | 19% | 13/16 | 81.2% [57.0-93.4] |
| Gemini 2.5 Pro | Q2 | 69.9 | 94% | 1/16 | 6.2% [1.1-28.3] |
| Gemini 2.5 Pro | Q3 | 86.2 | 100% | 0/16 | 0.0% [0.0-19.4] |
| Grok 4 | Q1 | 60.2 | 31% | 11/16 | 68.8% [44.4-85.8] |
| Grok 4 | Q2 | 65.0 | 88% | 2/16 | 12.5% [3.5-36.0] |
| Grok 4 | Q3 | 84.5 | 100% | 0/16 | 0.0% [0.0-19.4] |
| OpenAI O3 | Q1 | 52.0 | 12% | 14/16 | 87.5% [64.0-96.5] |
| OpenAI O3 | Q2 | 60.5 | 88% | 2/16 | 12.5% [3.5-36.0] |
| OpenAI O3 | Q3 | 85.5 | 94% | 0/16 | 0.0% [0.0-19.4] |
| Claude Sonnet 4 Thinking | Q1 | 51.4 | 12% | 14/16 | 87.5% [64.0-96.5] |
| Claude Sonnet 4 Thinking | Q2 | 61.5 | 88% | 2/16 | 12.5% [3.5-36.0] |
| Claude Sonnet 4 Thinking | Q3 | 87.3 | 100% | 0/16 | 0.0% [0.0-19.4] |

**Table N.16.2 Combined human Q1 critical errors and combined mean scores (bootstrap 95% CI).**

| **Quantity** | **Value [95% CI]** |
| --- | --- |
| Human Q1 critical-error rate (combined, 2 raters) | 1/32 = 3.1% [0.6–15.7] |
| Human Q1 critical errors on the 11 all-model-failure cases | 0/22 |
| Model Q1 critical-error rate (combined, 4 models) | 52/64 = 81.2% [70.0–88.9] |
| Human combined mean — Q1 | 68.3 [64.8–72.3] |
| Human combined mean — Q2 | 69.0 [62.4–75.2] |
| Human combined mean — Q3 | 93.4 [89.1–96.7] |

Interpretation. On the same cases, the human Q1 critical-error rate (3.1%; 0/22 on the all-model-failure subset) is far below the model rate (81.2%), with non-overlapping intervals, while on Q3 both humans and models are strong and no model produced a Dangerous Recommendation. This indicative within-task probe (enriched, n = 16) demonstrates that the Q1 items are answerable by competent human audiologists; it is framed as a targeted probe rather than a representative baseline and is not juxtaposed with the 35.4% population rate. Because it is a within-pipeline human-versus-model comparison, the contrast does not depend on the adjudicator being well-calibrated.

# **N.17 Short-Answer Reference-Answer Fidelity Audit**

To establish whether the low short-answer accuracy reflects model failure rather than a flawed answer key, a stratified sample of 210 research-article-derived short-answer items (enriched for all-model-failure plus comparison strata) was audited against the question and the full source abstract. A doctoral-level audiologist assigned a four-level fidelity label, and an independent LLM screen (Claude Opus 4.8) judged the same 210 items. An objective reference-grounding scan was computed over all 1,460 short-answer items, and a grading-fidelity re-judgment quantified automated-grader false-negatives. These fidelity labels are an LLM screen confirmed by a doctoral-level audiologist on the same items, not a multi-rater human gold standard.

**Table N.17.1 Reference-fidelity label distribution and reviewer agreement (210 items).**

| **Quantity** | **Value** |
| --- | --- |
| Human reviewer: Accurate / Defensible / Flawed / Indeterminate | 209 / 1 / 0 / 0 |
| LLM screen (Opus 4.8): Accurate / Defensible / Flawed / Indeterminate | 199 / 11 / 0 / 0 |
| Overall fidelity (Accurate or Defensible), Wilson 95% CI | 100% [98.2–100] |
| Human–LLM agreement: flawed vs not-flawed decision | 210/210 = 100% |
| Human–LLM agreement: four-level label | 198/210 = 94.3% |
| Reference-grounding scan over 1,460 items (mean / median) | 0.961 / 1.000 |
| Low-grounding items (<0.34); all tokenization artefacts of 1–2-word answers | 10 |

**Table N.17.2 Corrected short-answer accuracy after grading-fidelity re-judgment.**

| **Model** | **As graded** | **Re-graded (strict)** | **Delta (pp)** |
| --- | --- | --- | --- |
| Gemini 2.5 Pro | 30.00% | 32.33% | +2.33 |
| Grok 4 | 26.99% | 28.49% | +1.51 |
| OpenAI O3 | 23.90% | 26.37% | +2.47 |
| Claude Sonnet 4 Thinking | 22.81% | 24.38% | +1.58 |

*Note. Strict re-grade corrects automated-grader false-negatives on synonyms and unit/format variants permitted by the rubric (e.g., "30 seconds." vs "30 seconds"; "timbre perception" vs "timbre recognition"). Including borderline/defensible cases raises the best model to 33.22%. Model ranking is preserved.*

Worked example (Defensible, item SA_0694). The reference expands "PTW" as "Phonation Threshold Width" while the source abstract defines PTW as "phonation threshold power"; the substantive answer (PTW most sensitive among PTP/PTF/PTW) is directly supported by the abstract, so the naming inconsistency is labelled Defensible rather than Flawed.

Interpretation. Both the human reviewer and the independent LLM screen found zero flawed or indeterminate keys (100% fidelity), and the binary flawed/not-flawed decision agreed on all 210 items; disagreements were confined to Accurate-versus-Defensible severity. Because no key was flawed, removing flawed keys changes the short-answer score by 0 percentage points, and the most generous grading correction raises the best model only to 33.22%. The low short-answer scores therefore reflect genuine model failure, with any answer-key or grader artefact bounded at roughly 2–3 percentage points.

*Reporting note on kappa. Because both judges placed every audited item in the faithful category, the binary inter-rater kappa is undefined (zero marginal variance); raw agreement is reported instead, consistent with the high-agreement/low-kappa situation discussed for Q1 (Feinstein & Cicchetti).*
